# Targeting GNG4 inhibits tumor progression and restores enzalutamide sensitivity in prostate cancer by suppressing autophagy

**DOI:** 10.1038/s41419-026-08421-w

**Published:** 2026-01-28

**Authors:** Lei Chen, Jingyan Zhang, Yanshuo Hu, Xufeng Peng, Hong Wang, Binghua Chen, Jun Xia, Wei Xue, Chun-Wu Pan

**Affiliations:** 1 Department of Urology, Renji Hospital, School of Medicine, Shanghai Jiao Tong University, Shanghai, China; 2 Department of Urology & Nephrology, The First Affiliated Hospital of Ningbo University, Zhejiang, China; 3 Department of Pathology, Renji Hospital, School of Medicine, Shanghai Jiao Tong University, Shanghai, China

**Keywords:** Prostate cancer, Cancer therapeutic resistance

## Abstract

Prostate cancer (PCa) is the most prevalent malignancy among men worldwide. Advanced prostate cancer is characterized by aggressive progression, limited therapeutic response, and poor prognosis. Elucidating its oncogenic mechanisms may provide new opportunities for targeted intervention. Increasing evidence suggests that modulating cytoprotective autophagy represents a promising strategy for improving cancer treatment efficacy and overcoming drug resistance. Here, we identified the G protein subunit GNG4 as a crucial regulator of prostate cancer development. GNG4 expression was markedly elevated in advanced prostate cancer phenotypes and positively correlated with tumor survival, apoptosis, and migration. Further analysis demonstrated that GNG4 depletion suppressed autophagy and enhanced cellular sensitivity to enzalutamide. Mechanistically, GNG4 interacts with GNB1 to stabilize the downstream effector protein GNAI3 through the ubiquitination-proteasome pathway. These three distinct G protein subunits form a functional complex that regulates intracellular autophagy and subsequently influences the malignant behavior of prostate cancer. Furthermore, inhibition of autophagy or GNG4 knockdown significantly increased the antitumor efficacy of enzalutamide both in vitro and in vivo. Our findings identified GNG4 as a pivotal modulator of prostate cancer progression and proposed it as a promising therapeutic target to enhance the clinical response to enzalutamide.

**Figure:**
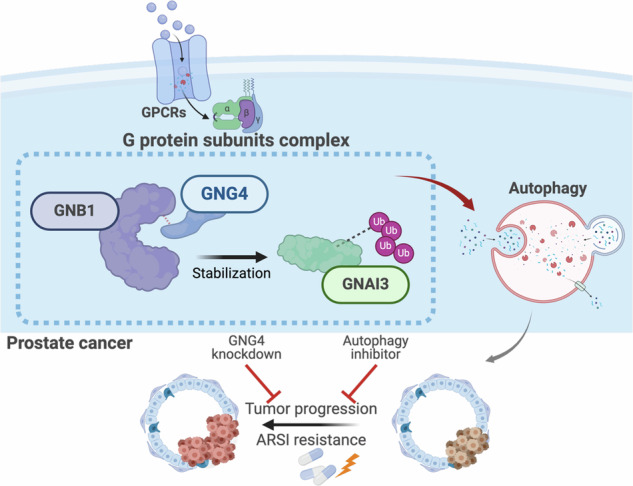
GNG4 interacts with GNB1 to stabilize GNAI3 via the ubiquitination-proteasome pathway, thereby activating autophagy. This process promotes prostate cancer progression and resistance to androgen receptor signaling inhibitors (ARSis). In contrast, GNG4 knockdown or pharmacological inhibition of autophagy restores ARSI sensitivity and suppresses tumor growth.

GNG4 interacts with GNB1 to stabilize GNAI3 via the ubiquitination-proteasome pathway, thereby activating autophagy. This process promotes prostate cancer progression and resistance to androgen receptor signaling inhibitors (ARSis). In contrast, GNG4 knockdown or pharmacological inhibition of autophagy restores ARSI sensitivity and suppresses tumor growth.

## Introduction

According to the latest cancer epidemiology data, prostate cancer (PCa) has become one of the most common cancer diagnoses and the second-leading cause of cancer death in men [1, 2]. Androgen deprivation therapy is currently a standard treatment for patients with advanced PCa. However, even following a period of time of treatment, most of the patients still inevitably face the stage of castration resistance [3–5]. New-generation androgen receptor signaling inhibitors (ARSI), such as bicalutamide, enzalutamide, and abiraterone, have demonstrated significant clinical benefits in the management of advanced PCa [6–9]. After the AFFIRM Phase III trial [8], the United States FDA approved using enzalutamide as further treatment for patients with castration-resistant PCa who have previously received docetaxel treatment. Applicability and therapeutic benefits of enzalutamide in advanced PCa have been addressed in numerous studies [10–12]. Nonetheless, we still face the challenge of overcoming treatment resistance, and the precise mechanisms involved remain to be elucidated. Hence, in order to improve the treatment response, further exploration on strengthening the effect of enzalutamide is necessary.

Nucleotide-binding protein (G protein) is a trimeric protein structure embedded in the cell membrane, comprising α, β, and γ subunits. It serves as a regulatory factor in transmembrane signaling pathways, mediating the transfer of information between the cell membrane and effector proteins. G protein acts as a “switch” that regulates intracellular effects, including cell metabolism, differentiation, secretion, proliferation, and death through multiple pathways [13]. G protein subunit gamma-4 (GNG4), as a member of γ chain of G protein, is essential for the G protein effector interaction [14]. It can combine with different α and β subunits to form a variety of G protein complexes, regulating downstream signaling pathways [15]. In recent years, several studies have identified GNG4 as a risk factor in adverse prognosis in tumors. Earlier research have also concluded that GNG4 exhibits elevated expression levels in multiple malignancies, including colon cancer, gastric cancer, gallbladder cancer, and lung adenocarcinoma [16–19]. The increased expression of GNG4 is associated with a higher level of immune cell infiltration within the tumor microenvironment, which serves as a guide for immunotherapy for bladder cancer [20]. In renal cell carcinoma, *GNG4* acts as a downstream gene regulated by von Hippel-Lindau (*VHL*) [21]. However, its role in PCa remains to be fully discovered.

Autophagy, a highly conserved metabolic process activated by stress responses, recycles aging proteins and organelles through lysosome-dependent degradation to regenerate energy [22]. It holds a significant position in cellular homeostasis, development, and apoptosis. Autophagy dysfunction is closely connected to the development of several major human diseases [23, 24]. Autophagy plays a crucial role in promoting cancer cell survival when cells are subjected to severe metabolic stress caused by chemotherapy, radiotherapy, starvation, oxidative stress, or growth factor deprivation. This is achieved by supplying essential nutrients and energy to these advanced-stage tumor cells and aiding immune evasion [25]. There is an increasing body of evidence suggesting that autophagy contributes greatly to the resistance mechanisms against different forms of chemotherapy. Jiang et al. showed that autophagy activation promotes aggressive development of triple-negative breast cancer cells and further induces taxanes chemoresistance [26]. Androgen receptor (AR) is essential in regulating the metabolic activities of prostate cells. ARSI therapy substantially suppresses AR-dependent biosynthesis and energy generation in PCa cells, leading to an acute metabolic stress microenvironment. Previous studies have shown elevated autophagy levels during short-term ARSI stress [27], and this overactivation persists until the castration-resistant stage [28]. Autophagy is deeply connected to metabolic reprogramming, particularly glucose and amino acid metabolism, sustaining the development and survival of PCa cells during ARSI treatments [27]. This cytoprotective autophagy is partially facilitated by AMPK pathway activation and the downregulation of mTOR signaling [29]. Targeting the AMPK pathway or inhibiting autophagy both demonstrated profound anti-tumor effects [28]. Mukha et al. discovered that reducing glutamine availability or disrupting its metabolic processes directly resulted in radiosensitization [30]. Under such circumstances, activation of ATG5-mediated autophagy appears to be a protective mechanism to counteract radiation-induced cell damage and death. Therefore, autophagy is regarded as a promising therapeutic target in PCa, and comprehending its mechanisms might help lead to a breakthrough in designing innovative therapeutic strategies.

Here, we identified GNG4 as a crucial regulator contributing to the aggressive malignant behavior and enzalutamide sensitization in PCa. Our results indicate that GNG4 is overexpressed in more advanced stages, and that its expression is associated with tumor growth and migration. GNG4 depletion suppressed the malignant phenotype of PCa cells by inhibiting autophagy. Most importantly, we elucidated the molecular mechanism by which GNG4 interacts with GNB1 (the G protein β subunit) to sustain GNAI3 (the G protein α subunit) expression. These components cooperatively regulate cytoprotective autophagy in a GNAI3-dependent manner, thereby influencing the cellular response to enzalutamide. Furthermore, in vivo experiments demonstrated that modulation of GNG4 expression sensitizes tumors to enzalutamide treatment. Collectively, our findings highlight GNG4 as a promising target for improving the therapeutic efficacy of enzalutamide in PCa.

## Materials and methods

### Cell culture, plasmids, and transfection

The DU145 and LNCaP prostate cancer cell lines used in this study were obtained from the Shanghai Institute of Bioscience and Cell Resources Center. The DU145 cell line was maintained in DMEM (Gibco), while the LNCaP cells were cultivated in RPMI-1640 medium (Gibco). Both cell types were grown in media containing 10% FBS (Gibco) and 100 U/ml penicillin-streptomycin (P/S, Thermo Fisher Scientific). All the cell cultivation was carried out in a humidified environment at 37 °C with 5% CO_2_.

Stable transfected cell lines were generated using a lentiviral packaging system. shRNAs targeting specific genes were constructed using the BR-V108 plasmid vector. The shRNA sequences, shGNG4#1: AGCCAGGAAAGCTGTGGAGCA; shGNG4#2: TGAAAGAGGGCATGTCTAATA; shGNG4#3: GCGGGAAGATCCTCTCATCAT. In compliance with the manufacturer’s protocol, lentiviral particles were harvested from HEK293T cells through transfection facilitated by Lipofectamine 2000 (Invitrogen). The efficiency of transfection was tested by fluorescence microscopy. Then, the puromycin was added in the cultural medium to maintain transfection efficiency. After transfection for 7-10 days, the efficiency was assessed through quantitative PCR and western blotting. To overexpression GNG4, its sequence was synthesized and subcloned into the *plv013-Flag-puro-CMV* vector, with empty vector as control.

### Public databases and dataset analysis

All the data of Prostate cancer adenocarcinoma (PRAD) in TCGA data platform was sourced from GTEx (commonfund.nih.gov/GTEx) website. This data contained clinical information of 496 prostate tumor samples. The transcriptome data were processed and normalized using FPKM for batch corrected mRNA gens expression. And all data were log2 transformed. To investigate variations in biological pathways, we utilized the GSEA tool (version 4.3.3) for performing gene set enrichment analysis. The selected gene sets comprised Hallmark, GO, and the KEGG, all sourced from the MSigDB database. To explore protein-protein interactions (PPIs), we utilized the STRING database (cn.string-db.org), inputting critical regulatory proteins to examine their role within the protein association network. PPI networks of GNG4 and GNAI3 were constructed on STRING v12.0 [31]. Cytoscape software was employed for data visualization, applying a threshold score of >0.4 while filtering out non-connected genes. The integrated bioinformatic platform UbiBrowser v2.0 (ubibrowser.ncpsb.org.cn) [32], was utilized to identify the potential human E3-substrate ubiquitin ligases of GNAI3 protein.

### Quantitative PCR (qPCR)

Cellular total RNA was extracted following the TRIzol Reagent (Ambion), adhering to the guidelines provided by the manufacturer. Reverse transcription was conducted using a specialized reaction mix (HiScript III RT SuperMix, Vazyme) to generate complementary DNA (cDNA). The amplification and quantification of gene expression were executed through a real-time PCR system utilizing a fluorescence-based detection reagent (Vazyme). The expression of target mRNA were determined using the 2^-ΔΔCq^ method.

### Western blotting

Protein extraction from cells was carried out using RIPA buffer, enriched with 1% PMSF and 1% protease inhibitors (Beyotime). Protein samples (≥10 μg) were subjected to separation via a polyacrylamide gel electrophoresis system (SDS-PAGE, 10%). To facilitate molecular weight estimation, a prestained protein marker (Thermo Fisher Scientific) was included. Following electrophoresis, the 0.45 μM PVDF membrane underwent blocking with a 5% milk solution prepared in TBST. Afterward, primary antibodies, appropriately diluted, were applied and incubated at 4 °C overnight. The following day, the membrane was treated with HRP-conjugated secondary antibodies at room temperature for one hour. Chemiluminescent signals were developed using BeyoECL reagent (Beyotime), and the protein bands were captured through a Tanon gel documentation system.

### Co-immunoprecipitation assay

All steps of co-immunoprecipitation assay were performed at 4 °C. Whole protein lysates were prepared from DU145 and LNCaP cells that had been transfected with GNG4-Flag and other relevant plasmids. To facilitate immune complex capture, 10 μL of Anti-Flag magnetic beads were incubated with IgG-specific antibodies (Beyotime). The extracted protein solutions were pre-washed with pre-cooled PBS before adding the pre-treated magnetic beads. These beads facilitated immune complex capture during a 12-h incubation with continuous rotation at 4 °C. The next day, 5 μL of the Protein A/G were added, and the incubation was continued for another two hours under rotation. After three rounds of washing, the beads were subjected to heat treatment at 95 °C for 10 min. Finally, the supernatant was analyzed via western blot using the specified primary antibodies.

### Wound healing assay

Transfected DU145 and LNCaP cells were plated in 6-well culture dishes until they reached 80–90% confluency. A sterile pipette tip was employed to generate a uniform scratch, and debris was washed with PBS. In order to limit proliferation effects, the culture medium was substituted with a serum-deprived version of RPMI-1640 or DMEM. Microscopic imaging was conducted at different time points to monitor how cells advanced into the gap region. The wound healing rates were subsequently analyzed by ImageJ software.

### Transwell assay

Cells were plated into the upper section of a dual-chamber transwell plates (Corning) containing 100 μL of non-serum medium. Meanwhile, the lower compartment was enriched with 10% FBS in 600 μL of culture medium. After 48 h of incubation, the cells were treated with 4% paraformaldehyde and then stained using a 0.05% crystal violet solution for 20 min. A sterile swab was used to remove the excess cells from the upper chamber. A light microscope was employed to assess migration efficiency and capture images of the transwell membrane. Five random fields from each insert were chosen, photographed, and processed using ImageJ software for analysis.

### Cell viability and colony formation assay

Tumor cell viability was measured using two methods: the CCK-8 assay and the Celigo cell counting assay, both performed according to the manufacturer’s protocols. Briefly, 2000 pretreated cells were distributed into a 96-well plate, cultured in a growth medium containing 10% FBS, and maintained for five days, with medium changes every two days. Regarding CCK-8 assay, 10 μL reagent was introduced into per well at designated time points, followed by incubation in darkness for two hours. Absorbance readings at 450 nm were then recorded using SkanIt Software v4.1 (Thermo Fisher Scientific). In parallel, the Celigo Image Cytometer was employed to track the count daily over a five-day period.

To assess colony formation, 1000 cells were seeded in six-well plates and cultured until colonies appeared. The colonies were fixed in 4% paraformaldehyde, stained with crystal violet, and then the colonies were counted. Clusters of more than 50 cells were considered a colony.

### Apoptosis and cell cycle analysis

Apoptosis was assessed using the Annexin V-APC/PI kit (eBioscience). Cells were washed, incubated with Annexin V at 4 °C, followed by addition of PI and a 10-min dark incubation. Flow cytometry analysis was performed with a BD FACS system, and data were analyzed using FlowJo software (version 10.10.0). Cell cycle distribution was analyzed by staining cells with a solution of PI (40×), RNase (100×), and PBS. Flow cytometry was used to determine the phase-specific cell distribution.

### Electron microscopy assays

Electron microscopy analysis was conducted after transfected cells were fixed overnight in glutaraldehyde, followed by washing and treatment with osmium tetroxide. Dehydration was carried out using a series of ethanol concentrations, and cells were then infiltrated with an embedding mixture and incubated. Ultra-thin sections were cut, stained with uranyl acetate and lead citrate, and examined using a transmission electron microscope (HT7800, Hitachi).

### Immunohistochemistry staining

Tissue samples were fixed in paraformaldehyde, embedded in paraffin, and sectioned into 4μm slices. After de-waxing, antigen retrieval was performed by heating in EDTA buffer. Endogenous peroxidase activity was blocked, and non-specific binding was minimized using horse serum. The sections were incubated with primary antibodies overnight, followed by secondary antibody treatment. The antigen detection was carried out with DAB staining, counterstained with hematoxylin, and mounted for imaging. Representative images were captured using an Olympus microscope.

### Protein-protein interaction interface analysis

To evaluate the interaction interface between GNG4 and GNB1protein, the protein docking software GRAMM-X (gramm.compbio.ku.edu) public server was used [33]. The protein sequence and 3-Dimensional structure of target proteins were sourced from a combination of databases, including the RCSB Protein Data Bank, UniProt database, and AlphaFold (alphafold.ebi.ac.uk). PyMOL molecular visualization system and PDBePISA were utilized for the study of protein interactions and subsequent visualization analysis.

### RNA sequencing

RNA sequencing was performed on DU145 cells transfected with either shCtrl or shGNG4, in triplicate. Total RNA was extracted using TRIzol and assessed for quality via the Agilent 2100 Bioanalyzer. Libraries were created with the TruSeq Stranded mRNA kit and sequenced on the Illumina HiSeq2000. The raw data were cleaned by removing low-quality reads and adapters, with paired-end 125 bp/150 bp reads generated. Differential expression was analyzed with a P-value cutoff of 0.05. Gene expression was quantified as FPKM with Cufflinks, and gene read counts were determined using HTSeq-count.

### Xenograft model in nude mice

Male BALB/c nude mice were randomly assigned to two or three or four groups for the study. DU145 or LNCaP cells were mixed with DMEM/RPMI-1640 and Matrigel (Corning) and injected into the right flank of 4-week-old male mice at a 200 μL volume per mouse. Tumor size was measured every five days over a four-week period using an electronic caliper, and Volume = 0.52 × Length × Width². A maximum tumor size of 2000 mm³ was maintained in accordance with institutional guidelines. Finally, tumors were harvested after mice euthanized, and their weight was measured with a precision scale. Enzalutamide (25 mg/kg), dissolved in 50% PEG-300 and 50% saline, was administered via oral gavage every other day.

### Ethics statement

Experiments involving animals were performed in compliance with the ethical standards established by the animal ethics committee of the Renji Hospital, Shanghai Jiao Tong University, China (Approval No. RA2021330).

### Statistical analysis

Statistical analyses were carried out using GraphPad Prism version 10.2.0 and SPSS Statistics version 27.0.1.0. All experiments were repeated in triplicate. Data are presented as mean ± SD. A two-tailed t-test equal variance was applied for comparisons. P-values < 0.05 were considered statistically significant (*P < 0.05, **P < 0.01, ***P < 0.001, and ****P < 0.0001).

## Results

### High expression of GNG4 is associated with advanced grade type in PCa

**Fig. 1:**
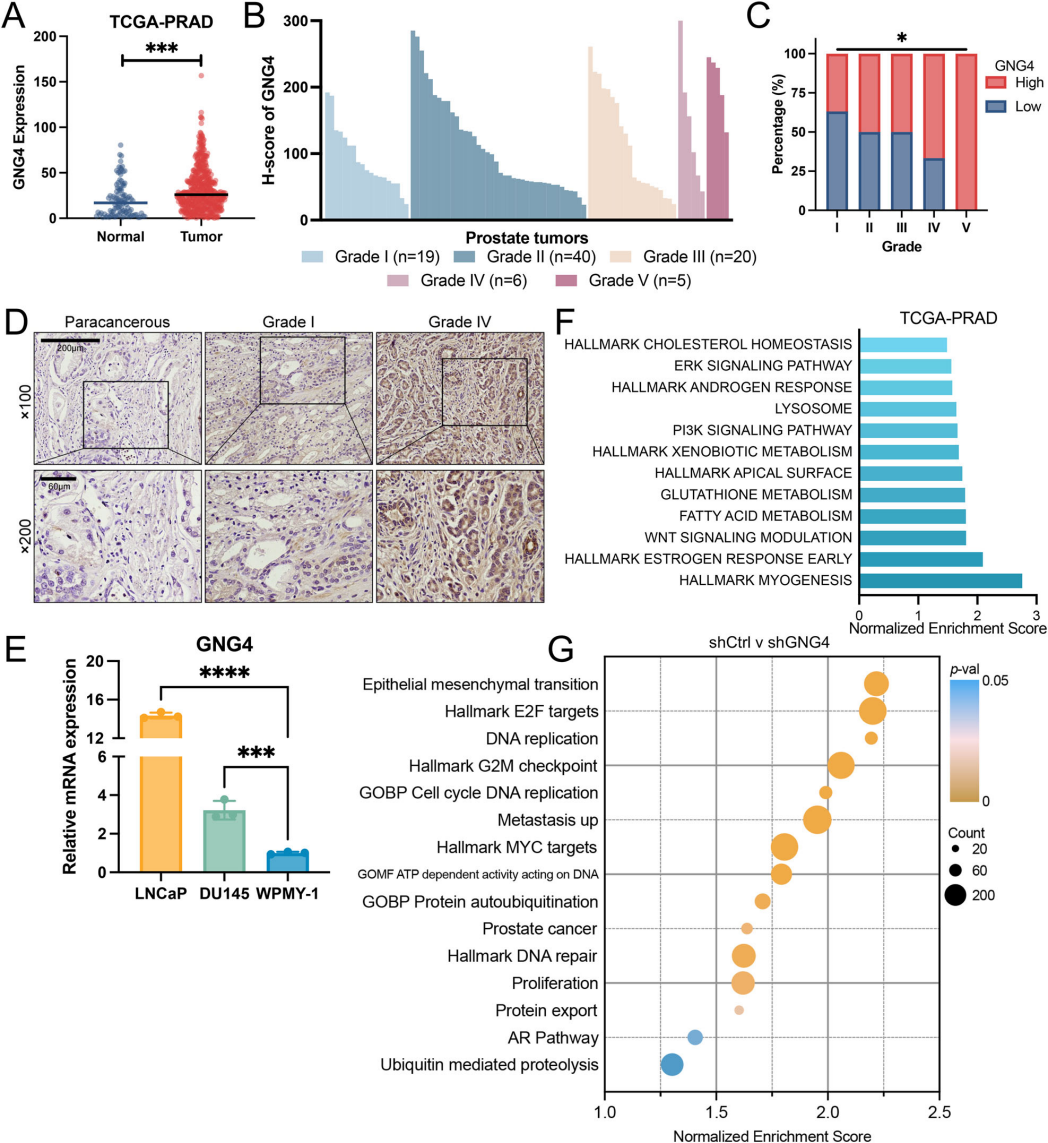
GNG4 expression is upregulated and associated with more aggressive grade type in prostate cancer. **A** Scatter plot showing the comparison of the GNG4 expression in normal prostate tissues and primary tumor tissues from the TCGA-PRAD dataset (normal [n = 100], tumor [*n* = 496]). **B** Waterfall diagram showing the immunohistochemistry staining H-score of GNG4 in prostate tumors and association with tumor grade. **C** Quantification bar chart of GNG4 Low and GNG4 High staining in indicated grade prostate cancer tissues (Grade I [*n* = 19], II [*n* = 40], III [*n* = 20], IV [*n* = 6], V [*n* = 5]). **D** Representative immunohistochemistry staining photographs of GNG4 in paracancerous tissue and various grade prostate cancer tissue (Scale bar, 200μm, 60μm). **E** qPCR analysis of GNG4 relative mRNA expression in prostate cancer cell lines and benign prostate hyperplasia cell. **F** Bar plot showing the gene set enrichment analysis of Hallmark and KEGG pathway collection in GNG4 high-expression group from TCGA-PRAD database. **G** Gene set enrichment analysis positively enriched in DU145 cells transcriptome sequencing (shCtrl vs. shGNG4). Results are listed by normalized enrichment score.

To identify the critical drivers of PCa, we collected paired tumor and adjacent normal tissues from ten PCa patients who underwent radical prostatectomy. Transcriptomic and differential gene expression profiling analyses revealed several candidate genes that were significantly upregulated in tumor samples (Supplementary Fig. S1A). To assess their functional relevance, the effects of these genes on cell viability were assessed examined CCK-8 assays (Supplementary Fig. S1B). Among them, GNG4 was identified as the most prominent candidate, demonstrating a marked reduction in tumor cell proliferation following its knockdown. Based on these results, GNG4 was selected for further investigation to elucidate its mechanistic role in PCa. We next examined the expression pattern of GNG4 in benign and malignant prostate tissues. Microarray-based expression analysis of the TCGA-PRAD dataset, comprising 100 normal and 496 tumor tissues, revealed that GNG4 expression was significantly upregulated in primary PCa samples (Fig. 1A). To validate the results obtained from the public dataset, we examined GNG4 expression by immunohistochemistry (IHC) in normal and malignant human prostate tissues. The staining results and clinicopathological characteristics of the patients are summarized in Supplementary Table 1–3. Tumor sections were evaluated and graded according to the ISUP grade standard. Our findings indicated a marked increase of GNG4 expression in higher-grade PCa tissues (Fig. 1B, C). IHC staining further demonstrated that GNG4 levels were markedly elevated in tumor tissues, whereas its expression in normal prostate tissues was nearly undetectable (Fig. 1D). Additionally, we also evaluated GNG4 expression across a panel of cell lines. qPCR analysis showed that GNG4 mRNA levels were significantly higher in LNCaP and DU145 PCa cell lines compared to the normal prostatic epithelial cell line WPMY-1 (Fig. 1E).

To gain deeper insights into the role of GNG4 in PCa, we analyzed the TCGA-PRAD dataset to identify biological pathways enriched in association with GNG4 expression. The analysis revealed that the most enriched hallmark and KEGG pathways in GNG4^High^ compared with GNG4^Low^ samples were primarily involved in fatty acid and glutathione metabolism, lysosome function, androgen response, as well as the WNT, PI3K, and ERK signaling pathways (Fig. 1F). Notably, depletion of GNG4 resulted in marked enrichment of several oncogenic pathways according to hallmark and Gene Ontology analyses, including cell cycle, metastasis, DNA repair, proliferation, epithelial-mesenchymal transition, E2F, MYC, and AR pathways (Fig. 1G). These findings suggest that GNG4 plays a pivotal role in driving PCa progression through multiple oncogenic pathways.

### GNG4 is essential for the development of PCa

**Fig. 2:**
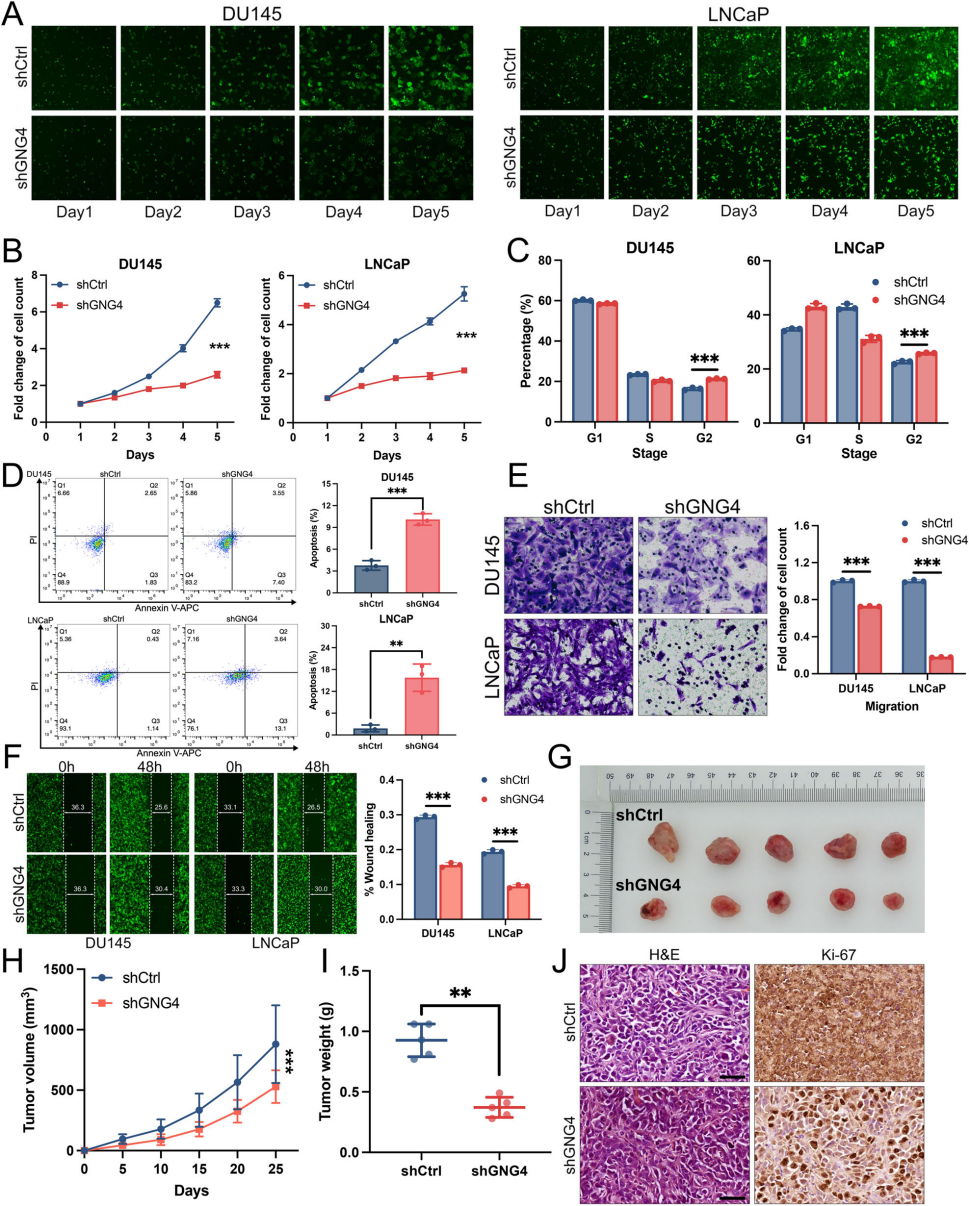
GNG4 is essential for the growth and tumor progression of prostate cancer. **A** Representative fluorescence cell images of DU145 and LNCaP cells (shCtrl and shGNG4) at indicated time points. Images were captured by a Celigo Image Cytometer in 5 days. **B** Cell viability of DU145 and LNCaP cells (shCtrl and shGNG4) were determined by Celigo cell counting assay at indicated time points. **C** Quantitative analysis of cell cycle in DU145 and LNCaP cells (shCtrl and shGNG4) were used by flow cytometric assays. **D** Cell apoptosis level of DU145 and LNCaP cells transfected with shCtrl and shGNG4 was determined by flow cytometry. Representative graph (left) and quantitative analysis (right). **E** Transwell assays were conducted to evaluate the effect of GNG4 on the migratory abilities of DU145 and LNCaP cells. **F** Representative images (left) and statistical results (right) of the wound healing assays in DU145 and LNCaP cells (shCtrl and shGNG4). Gap closure (%) = (0–48 h distance)/0 h distance. **G** The photograph showing DU145 xenografts in nude mice with shCtrl and shGNG4 (*n* = 5, per group). **H** Tumor volume of each group was measured once a week at indicated time points. **I** Tumor weight was measured when sacrificed. **J** Representative H&E and Ki-67 staining images in xenografts (Scale bar, 50μm).

To assess whether GNG4 acts as a crucial regulator of PCa, we examined the effects of GNG4 knockdown through both in vitro/vivo analyses. Initially, shRNA constructs were used to suppress GNG4 expression in DU145 and LNCaP cells, and knockdown efficiency was validated by qPCR and western blot analysis (Supplementary Fig. S1C–E). Our results from the Celigo cell counting assay demonstrated that depletion of GNG4 significantly inhibited cell proliferation in both DU145 and LNCaP cells compared with the control group (Fig. 2A, B). Given the observation that GNG4 may participate in the cell cycle progression and DNA replication, we conducted flow cytometric analysis and revealed a significant accumulation of cells in the G2 phase following GNG4 knockdown (Fig. 2C). Moreover, GNG4 downregulation markedly increased apoptosis (Fig. 2D), indicating that GNG4 might influence cell growth by modulating both the cell cycle and apoptosis processes. To further test whether GNG4 affects other malignant phenotypes, we performed migration and wound healing assays, which indicated that GNG4 knockdown markedly significantly impaired cell migration and wound healing capacity (Fig. 2E, F).

To evaluate the effect of GNG4 in tumor growth in vivo, DU145 cells with or without GNG4 knockdown were injected into the flanks of BALB/c nude mice to establish xenograft tumors. The results demonstrated that GNG4 depletion significantly reduced tumor growth and tumor weight (Fig. 2G–I). Consistently, IHC staining analysis revealed that Ki-67 expression was substantially higher in the negative control group than in the GNG4 knockdown group. (Fig. 2J). Collectively, these findings indicate that GNG4 plays an essential role in prostate cancer progression by promoting tumor cell proliferation and migration, supporting its function as an oncogenic driver in PCa.

### Modulation of GNG4 alters autophagy level and enzalutamide sensitivity

**Fig. 3:**
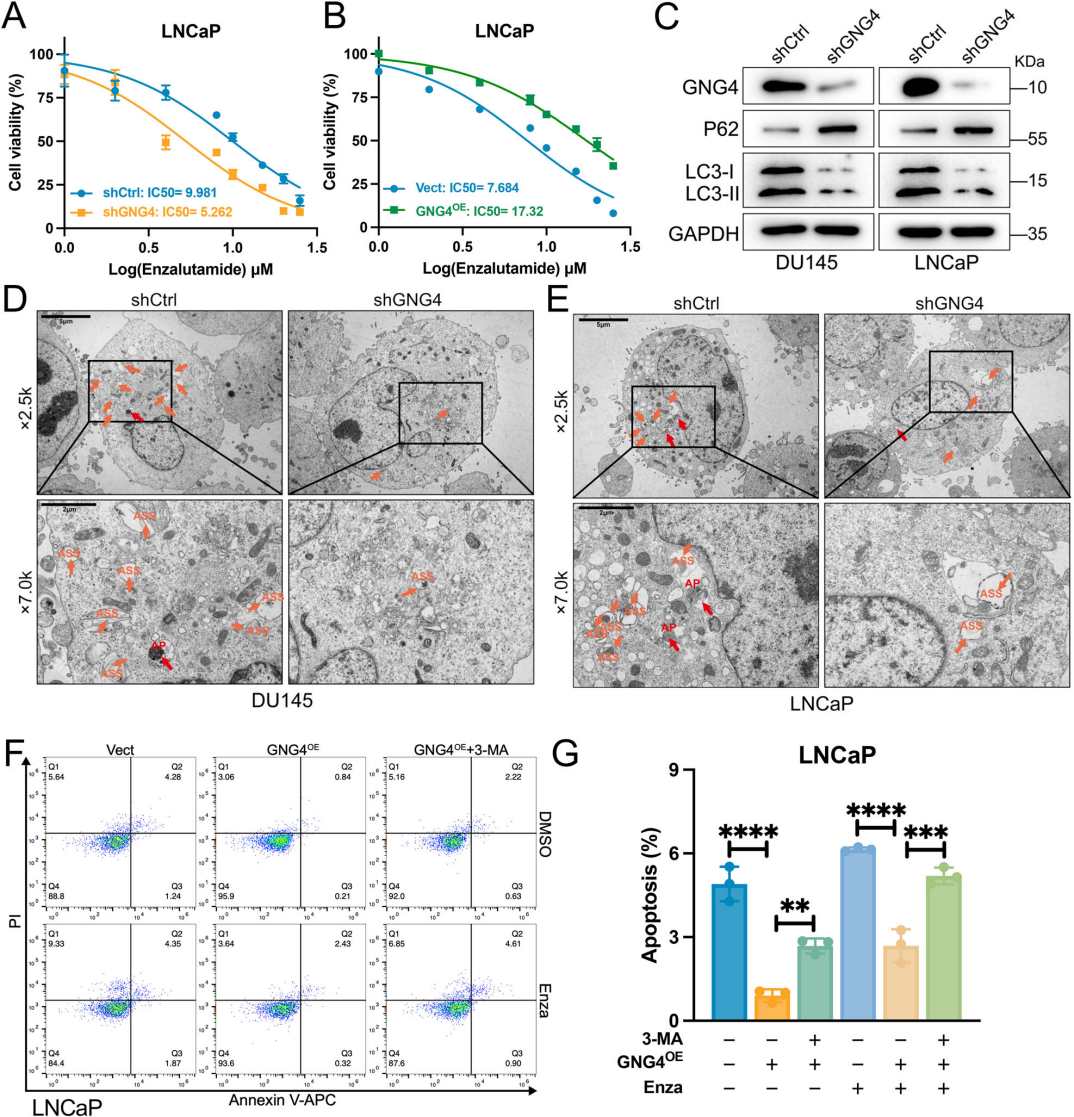
Modulation of GNG4 alters autophagy level and enzalutamide sensitivity. **A** Cell viability of LNCaP/shCtrl and LNCaP/shGNG4 treated with increasing concentration of enzalutamide. **B** Cell viability of LNCaP/Vect and LNCaP/GNG4^OE^ treated with increasing concentration of enzalutamide. CCK-8 assays were performed and the IC50 of enzalutamide were calculated. **C** Western blot analysis of autophagy related protein expression in DU145 and LNCaP cells (shCtrl and shGNG4). **D, E** Representative electron microscopy images of autophagic vacuoles in DU145 and LNCaP cells (shCtrl and shGNG4). Scale bar, 5μm; 500 nm. Orange arrows depict autolysosomes (ASS), red arrows depict autophagosomes (AP). **F** Representative flow plots and **G** quantification of the cell apoptosis level in LNCaP cells transfected with Vector, GNG4 overexpression (GNG4^OE^), or GNG4^OE^ plus 3-Methyladenine (3-MA). LNCaP cells were treated with 17μm Enza or DMSO for 24 h. Enza, enzalutamide.

Given that resistance to androgen deprivation therapy is a key feature of tumor progression, we next examined whether GNG4 influences the response to enzalutamide. Cell viability assays showed that knockdown of GNG4 rendered LNCaP cells more sensitive to enzalutamide (Fig. 3A), whereas overexpression of GNG4 reduced drug sensitivity (Fig. 3B). These results suggest that GNG4 deficiency enhances cellular responsiveness to enzalutamide treatment.

Autophagy is known to modulate tumor sensitivity to various chemotherapeutic agents, and previous studies have reported that increased autophagic activity is associated with the development of castration resistance [34]. However, the relationship between GNG4 and autophagy remains poorly defined. To further clarify this connection, we conducted western blot analysis after GNG4 depletion. The results showed a significant reduction in LC3, a key autophagy-related protein, in GNG4-deficient cells compared with controls. Additionally, the expression of the autophagy receptor P62 was elevated, indicating a suppression of autophagic activity (Fig. 3C). Consistently, transmission electron microscopy demonstrated that the GNG4 knockdown reduced the number of autophagic vacuoles (Fig. 3D, E).

Overexpression of GNG4 suppressed apoptosis and promoted cell proliferation in both LNCaP and DU145 cells under basal conditions (Fig. 3F, G, Supplementary Fig. S1F–I). In LNCaP cells treated with enzalutamide, apoptosis was markedly increased, but this effect was partially inhibited by GNG4 overexpression (Fig. 3F, G). Nevertheless, these effects were significantly attenuated when autophagic process was blocked by 3-methyladenine (3-MA). Together, these findings indicate that GNG4 regulates autophagy in PCa, thereby modulating enzalutamide sensitivity and contributing to malignant cell growth.

### GNG4 stabilizes GNAI3 by binding to GNB1 protein

**Fig. 4:**
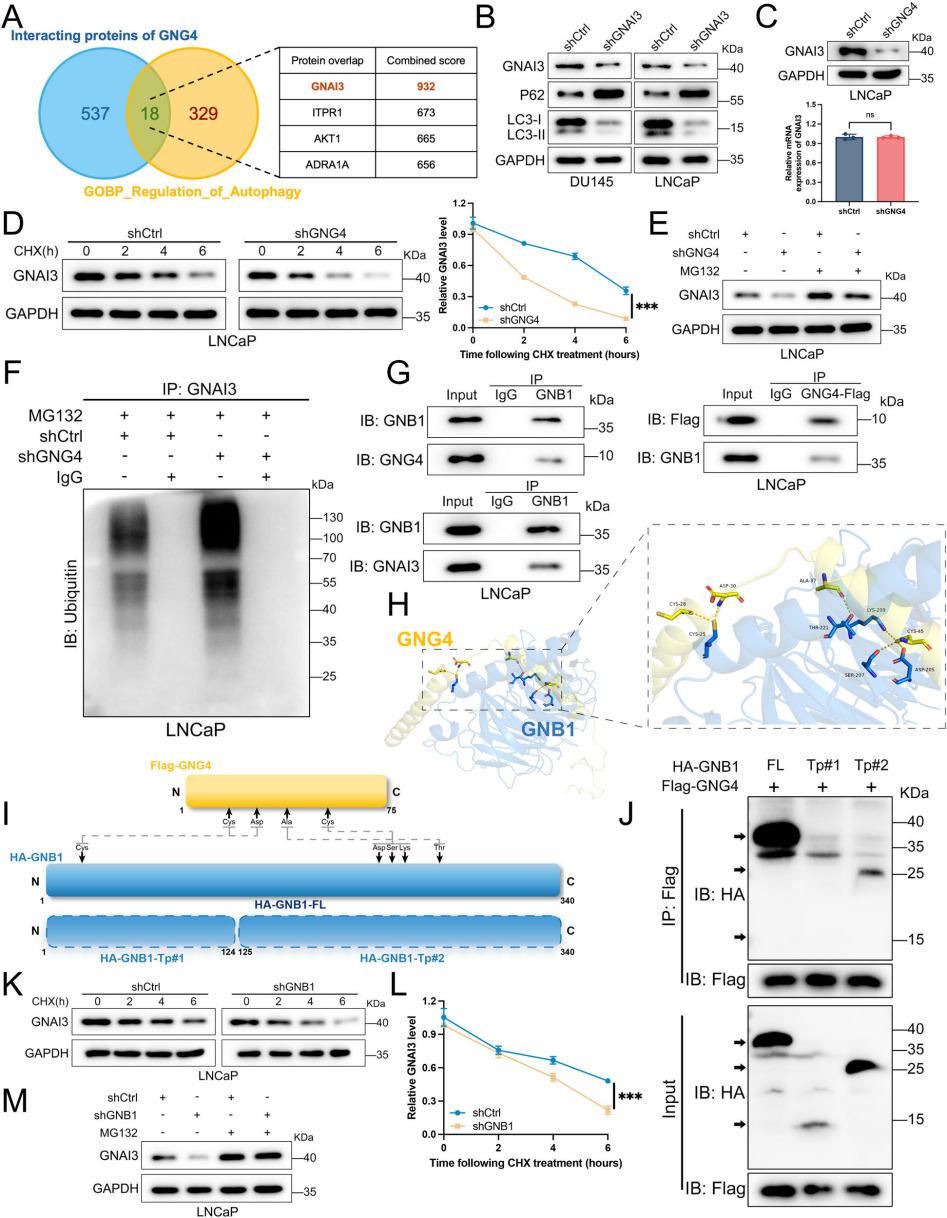
GNG4 stabilizes GNAI3 by binding to GNB1 protein. **A** Venn diagram (left) showing the overlap of predicted interacting proteins of GNG4 and GOBP_Regulation_of_Autophagy set. Top candidates were listed at the table (right) attached with combined score. **B** Western blot analysis of autophagy related protein expression in DU145 and LNCaP cells (shCtrl and shGNAI3). **C** qPCR and western blot analysis of GNAI3 mRNA and protein expression in the LNCaP cells following the GNG4 knockdown. **D** LNCaP cells stably knockdown GNG4 or negative control were treated with cycloheximide (CHX, 50 μg/ml) at indicated time points by western blot analysis (left). The GNAI3 protein expression level was quantified, and the plot was presented (right). **E** LNCaP/shCtrl or LNCaP/shGNG4 cells were treated with 10μmol/L MG132 for 4 h, the GNAI3 protein level was detected by western blot analysis. **F** Western blot analysis of ubiquitinated GNAI3 immunoprecipitated from LNCaP cells with or without GNG4 knockdown. **G** The interaction between GNG4 and GNB1 using anti-GNB1 antibody in LNCaP cells was examined by immunoprecipitation (left). LNCaP cells were transfected Flag-tagged GNG4 as indicated. Cell lysates were immunoprecipitated with Flag antibodies, and cell immunoprecipitates were immunoblotted with Flag or GNB1 antibodies (right). The interaction between GNB1 and GNAI3 using anti-GNB1 antibody in LNCaP cells was examined by immunoprecipitation (down). **H** Structure-based protein interaction interface analysis between GNG4 and GNB1. Cartoon represents the predicted GNG4/GNB1 complex structure, where the interaction hotspot residues are labeled. **I** Schematic diagram of truncations of GNB1 for co-immunoprecipitation experiments, and predicted interaction sites on GNB1 with GNG4. **J** Co-immunoprecipitation of GNG4 and GNB1 truncation proteins in HEK293T cells transfected with Flag-GNG4 and different HA-GNB1 (*FL: full length, Tp: truncation protein*) plasmids. **K** LNCaP cells (shCtrl or shGNB1) were treated with CHX at indicated time points by western blot analysis. **L** The GNAI3 protein expression level was quantified, and the plot was presented. **M** LNCaP/shCtrl or LNCaP/shGNB1 cells were treated with MG132 for 4 h, the GNAI3 protein level was detected by western blot analysis.

The results described above indicate a strong association between GNG4 and autophagic activity, as alterations in GNG4 expression directly affect intracellular autophagy. We next sought to elucidate the underlying molecular mechanisms. Based on the predicted protein set that interacted with GNG4 obtained from the STRING database and GOBP_Regulation_of_Autophagy dataset, we identified 18 potential proteins by intersecting the two sets (Fig. 4A). Among these, GNAI3 (G protein subunit alpha-3) showed the highest combined score. Previous reports have also implicated GNAI3 in autophagy regulation in several disease contexts [35, 36]. Consequently, GNAI3 was selected as a potential downstream effector of GNG4 for further investigation. Western blot analysis revealed a decrease in LC3 expression and a concomitant increase in P62 levels following GNAI3 knockdown (Fig. 4B), consistent with the effects observed after GNG4 depletion (Fig. 3C). Although alterations in GNG4 expression did not change the mRNA levels of GNAI3, a significant reduction in GNAI3 protein abundance was detected upon GNG4 knockdown (Fig. 4C, Supplementary Fig. S2A). Hence, we hypothesized that GNG4 may regulate GNAI3 expression through post-transcriptional mechanisms.

Next, we carried out cycloheximide (CHX) chase assays to evaluate the stability of GNAI3 protein. The results revealed that depleting GNG4 markedly reduced the amount of GNAI3 protein levels over time (Fig. 4D, Supplementary Fig. S2B, C). Treatment with MG132, a proteasome inhibitor, reversed the GNG4 knockdown-induced decrease in GNAI3 abundance (Fig. 4E, Supplementary Fig. S2D), indicating that GNG4 regulates GNAI3 stability through a proteasome-dependent mechanism. To further examine whether this regulation involves ubiquitination, we carried out ubiquitination assays. GNG4 knockdown in LNCaP and DU145 cells led to a substantial increase in endogenous ubiquitination of GNAI3 compared to negative control group (Fig. 4F, Supplementary Fig. S2E). Moreover, our data revealed that GNG4-mediated ubiquitin linkage was primarily associated with Ub-K48 rather than Ub-K63 (Supplementary Fig. S2F, G). Collectively, these findings indicate that GNG4 regulates GNAI3 protein stability through the ubiquitination-proteasome pathway.

To investigate how GNG4 regulates GNAI3, we utilized UbiBrowser v2.0 to predict potential ubiquitin ligases of GNAI3 and intersected these with the set of proteins predicted to interact with GNG4 in the STRING database (Supplementary Fig. S2H). GNB1 (G protein subunit beta-1), which participates in transmembrane signaling, was identified as a key candidate in this regulatory network. Altering GNG4 expression did not affect GNB1 protein content (Supplementary Fig. S2I).

Endogenous and semi-exogenous co-immunoprecipitation (co-IP) assays confirmed that GNB1 interacted with both GNG4 and GNAI3 (Fig. 4G, Supplementary Fig. S2J). To further characterize the interaction interface, we analyzed the interaction domains of GNG4/GNB1 using the UniProt and AlphaFold protein structure databases and constructed a three-dimensional structural model of the GNG4/GNB1 complex. Docking simulation predicted that the amino acids (AAs) Cys-28, Asp-30, Ala-37, and Sys-45 of GNG4, together with AAs Cys-25, Thr-221, Asp-205, and Ser-207 of GNB1, were responsible for their interactions (Fig. 4H). To validate the predicted binding region, full-length (FL) and truncated protein (Tp) variants of HA-tagged GNB1 plasmids were co-transfected with Flag-tagged GNG4 plasmids into HEK293T cells (Fig. 4I). Co-IP analysis showed that the full-length and truncated variant #2 (Tp#2, 125-340AAs) of GNB1 interacted with GNG4 (Fig. 4J), whereas truncated variant #1 (Tp#1, 1-124AAs) did not, suggesting the binding domain of GNB1 responsible for GNG4 interaction lies within amino acids 125–340.

Subsequently, we found that GNB1 knockdown led to reduced protein stability of GNAI3 (Fig. 4K, L, Supplementary Fig. S2K). Consistent with the results of GNG4 depletion, GNB1 modulated GNAI3 stability via a proteasome-dependent mechanism (Fig. 4M, Supplementary Fig. S2L). Together, these results suggest that GNG4 stabilizes the GNAI3 by directly combining with the mediator GNB1.

### GNG4 promotes malignant progression of PCa relying on GNAI3

**Fig. 5:**
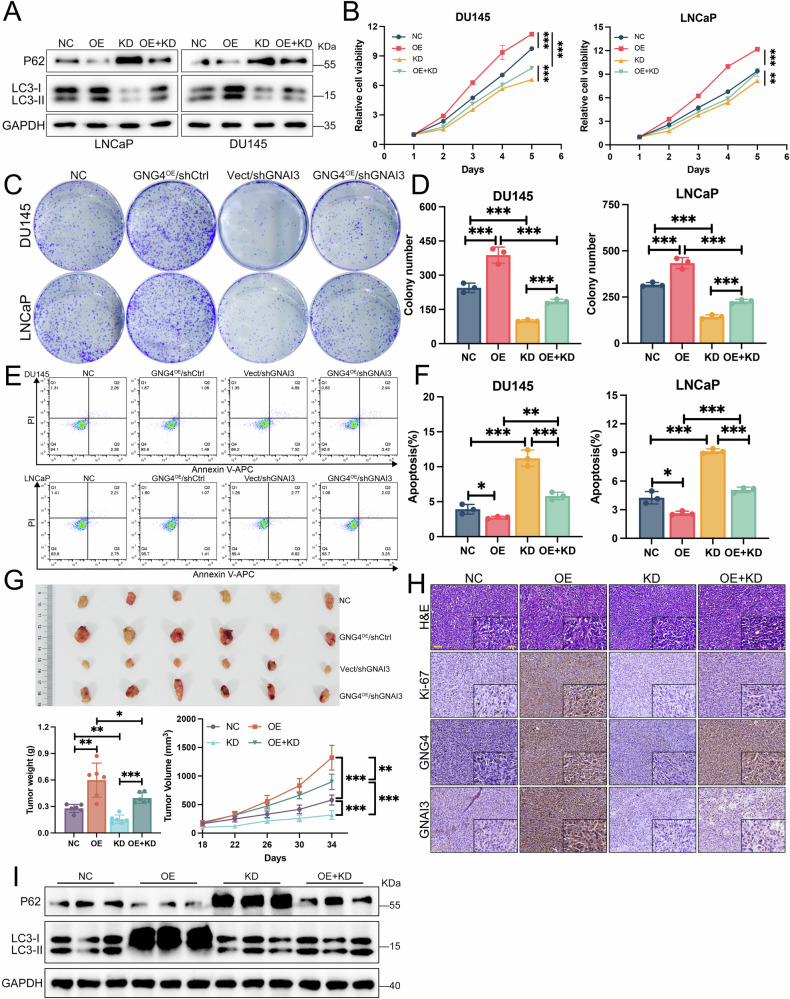
GNG4 relies on GNAI3 to regulate the malignant progression of prostate cancer. **A** Western blot analysis of autophagy related protein levels in LNCaP and DU145 cells transfected with vector, GNG4, and/or shGNAI3 plasmids as indicated. *NC: negative control, Vector plus shCtrl; OE: GNG4 overexpression plus shCtrl; KD: Vector plus GNAI3 knockdown, OE* + *KD: GNG4 overexpression plus GNAI3 knockdown*. **B** Effect of GNG4 overexpression and/or GNAI3 knockdown on DU145 and LNCaP cell viability were examined by CCK-8 assays. **C** Representative images of colony formation assays and **D** quantification results in DU145 and LNCaP cells with different treatment as indicated. **E** Cell apoptosis level of DU145 and LNCaP cells transfected with GNG4 and/or shGNAI3 was determined by flow cytometry. Representative graph and **F** quantitative analysis results. **G** Xenograft tumors of DU145 cells with various plasmids transfection formed in nude mice (*n* = 6 mice per group). Tumor weight was measured at the end of the experiment. Tumor growth curves were recorded after subcutaneous injection. **H** Representative images of H&E staining and immunohistochemistry for Ki-67, GNG4, and GNAI3 staining in xenografts. Scale bar, 50μm. **I** Western blot analysis of autophagy-related protein expression in DU145 xenografts.

The findings above support the conclusion that GNAI3 functions acts as a key downstream effector of GNG4. We next examined investigated whether GNAI3 contributes to GNG4-induced tumor progression. GNG4 expression was elevated in LNCaP and DU145 cells, whereas GNAI3 was silenced using shRNA. Western blotting analysis revealed that GNAI3 knockdown inhibited the autophagy activation induced by GNG4 overexpression (Fig. 5A). Rescue experiments further demonstrated that GNAI3 deficiency partially reversed the enhanced cell proliferation caused by GNG4 (Fig. 5B–D). In addition, apoptosis assays revealed that the GNAI3 knockdown restored the reduced apoptosis rate observed in PCa cells transfected with the GNG4 overexpression plasmid (Fig. 5E, F). These results indicated that GNG4 promotes malignant progression in PCa through GNAI3-mediated autophagy.

To assess the relevance of the GNG4/GNAI3 axis to tumor growth in vivo, DU145 cells with GNAI3 knockdown and/or GNG4 overexpression were subcutaneously implanted into male nude mice. Tumor growth was monitored for approximately five weeks. Tumors with elevated GNG4 expression exhibited significantly increased growth rates and greater weight accumulation compared with controls, whereas GNAI3 knockdown mitigated the accelerated tumor growth rate caused by GNG4 overexpression (Fig. 5G). IHC staining further confirmed that GNG4, GNAI3, and the proliferation marker Ki-67 were highly expressed in tumors overexpressing GNG4 but were reduced upon GNAI3 silencing (Fig. 5H). Consistent with the in vitro findings, Western blot analysis of xenograft tumor tissues confirmed that GNG4 overexpression enhanced autophagic activity in vivo, as evidenced by increased LC3-II and decreased P62 levels. However, this GNG4-induced autophagy was markedly attenuated when GNAI3 was silenced (Fig. 5I). Collectively, these findings provide evidence that autophagy regulated by GNAI3 plays a crucial role in GNG4-driven tumor progression.

### GNG4 modulates autophagy to enhance enzalutamide sensitivity in PCa

**Fig. 6:**
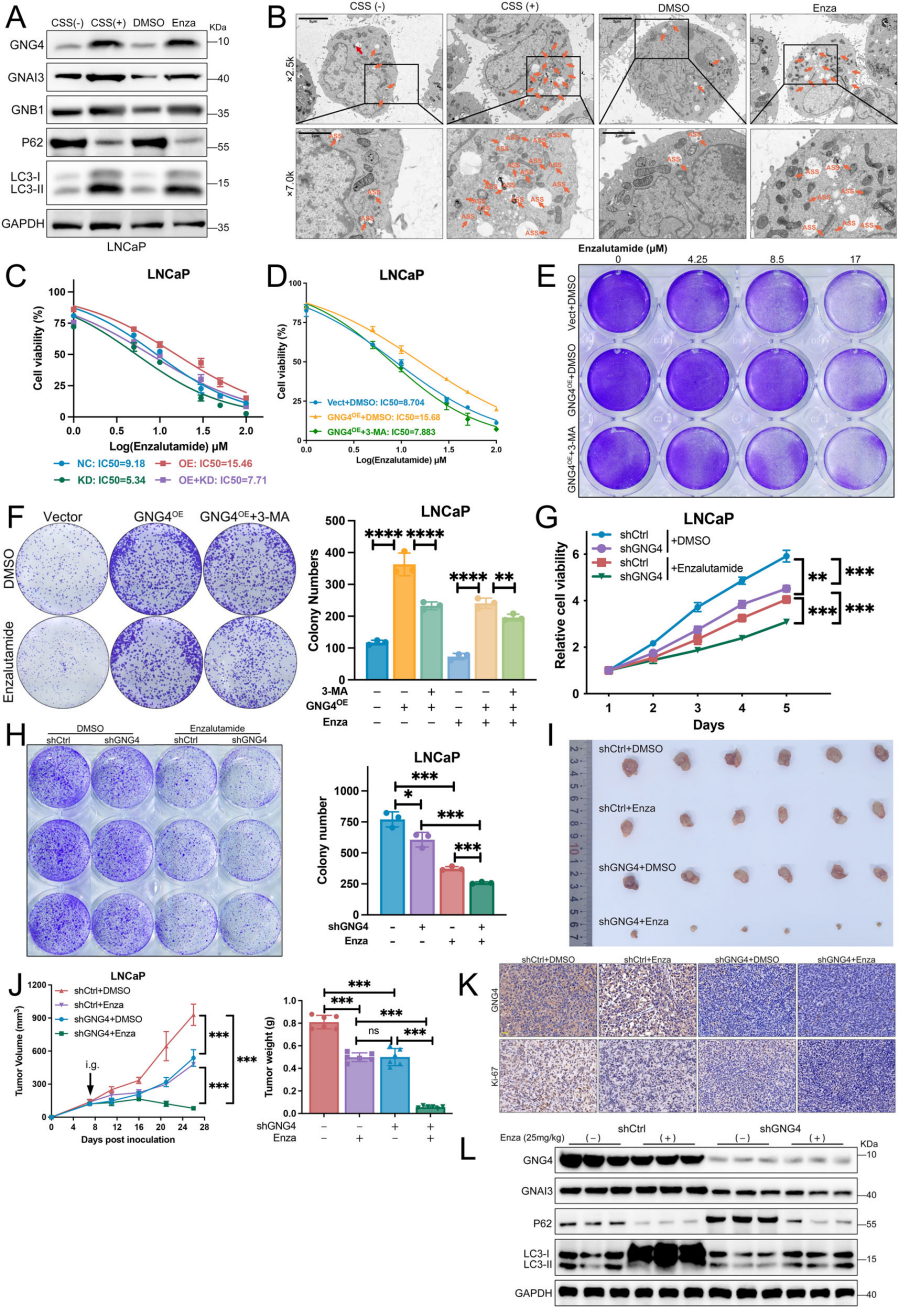
GNG4–autophagy axis regulates the response to enzalutamide in prostate cancer. **A** Western blot analysis of GNG4, GNB1, GNAI3, and autophagy-related protein expression in LNCaP cells under different treatments: normal medium (CSS − ), charcoal-stripped serum (CSS + ) medium, DMSO, and Enzalutamide (10 μM). **B** Representative transmission electron microscopy images of autophagic vacuoles in LNCaP cells. Scale bar, 5μm; 500 nm. Orange arrows depict autolysosomes (ASS), red arrows depict autophagosomes (AP). **C** Cell viability curve for LNCaP cells with different plasmids transfection treated with various concentration of enzalutamide. **D** Cell viability of LNCaP/Vect, GNG4^OE^, and GNG4^OE^ + 3-MA treated with various concentration of enzalutamide. **E** Crystal violet cell viability assay showing LNCaP cells with various treatments (Vector, GNG4^OE^, GNG4^OE^ + 3-MA) under increasing concentrations of enzalutamide. **F** Colony formation assays were performed to examine the cell proliferation of LNCaP cells subjected to various treatments with enzalutamide or DMSO. Representative photographs (left) and quantification bar chart (right) were shown. **G** The dose-response curve CCK-8 assay illustrates cell viability in LNCaP cells treated with increasing concentrations of enzalutamide under various conditions (shCtrl or shGNG4). **H** Colony formation assays were performed to evaluation the viability of LNCaP cells (shCtrl or shGNG4) treated with either DMSO or enzalutamide. Representative photographs (left) and quantification bar chart (right) were shown. **I** Xenograft tumors derived from LNCaP cells (shCtrl or shGNG4) established in nude mice treated with DMSO or enzalutamide (*n* = 6 per group, 25 mg/kg, [i.g.] intragastric). **J** Tumor volumes were recorded throughout the experiment (left), and tumor weights were measured at the endpoint (right). **K** Representative images of immunohistochemistry staining for GNG4 and Ki-67 staining in xenografts. Scale bar, 50μm. **L** Western blot analysis of GNG4, GNAI3, and autophagy-related protein expression in xenograft tumors.

To explore whether autophagy is activated upon androgen receptor pathway inhibition, LNCaP cells were cultured in charcoal-stripped serum (CSS) medium or treated with enzalutamide for 48 h. Western blot and transmission electron microscopy analyses revealed a marked increase in autophagy-related protein levels and the accumulation of autophagic vacuoles within the cells (Fig. 6A, B). In addition, the expression of GNG4 was elevated under these stress conditions(Fig. 6A), suggesting that GNG4 may participate in the adaptive autophagic response triggered by AR signaling blockade.

Building on these findings, we next investigated whether alterations in GNG4 expression influence the responsiveness of PCa cells to enzalutamide. Inhibition of autophagy markedly enhanced cellular sensitivity to the drug. Furthermore, manipulating the GNG4/GNAI3 axis altered autophagy levels, which consequently affected the sensitivity of PCa cells to enzalutamide (Fig. 6C).

Consistently, dose–response analyses showed that elevated GNG4 expression substantially increased the IC50 value of enzalutamide (15.68 μM), compared with control groups (IC50 = 8.704 μM). Treatment with the autophagy inhibitor 3-MA partially restored drug sensitivity in GNG4-overexpression cells (IC50 = 7.883 μM), indicating that suppression of GNG4-induced autophagy enhances cellular responsiveness to enzalutamide (Fig. 6D). Crystal violet viability and colony formation assays further supported these findings, showing that GNG4 overexpressing cells exhibited higher viability and formed more colonies under enzalutamide treatment, whereas the addition of 3-MA markedly reduced colony formation in GNG4 overexpressed LNCaP cells. (Fig. 6E, F). To further assess whether reducing GNG4-regulated autophagy could enhance enzalutamide sensitivity, we performed cell viability assays following drug treatment. CCK-8 analysis demonstrated that GNG4 knockdown cells exhibited significantly lower viability than control cells in response to enzalutamide (Fig. 6G). Similarly, colony formation assays showed that the GNG4 knockdown group produced markedly fewer colonies after enzalutamide treatment, indicating an improved therapeutic response due to GNG4 suppression (Fig. 6H). These results reinforce the notion that GNG4-mediated autophagy is a key determinant of cellular sensitivity to enzalutamide in prostate cancer.

To further assess the translational relevance of our findings, we evaluated the in vivo effects of GNG4 modulation on the therapeutic response to enzalutamide. LNCaP cells with or without GNG4 knockdown were subcutaneously injected into nude mice, followed by oral administration of DMSO or enzalutamide (25 mg/kg). Both GNG4 silencing and enzalutamide treatment suppressed tumor growth, while their combination achieved the strongest inhibition, as shown by the smallest tumor volumes and lowest weights (Fig. 6I, J). No significant weight loss was observed in any of these mouse models (Supplementary Fig. S2M). Immunohistochemistry revealed markedly reduced Ki-67 expression in the combination group (Fig. 6K). Western blot analysis of tumor tissues showed decreased reduced autophagy activity, indicated by P62 accumulation and lower LC3-I/II expression (Fig. 6L). This synergistic effect may be attributed to the suppression of GNG4-mediated autophagy, which enhances the sensitivity of tumors to enzalutamide treatment. These findings reinforce the translational relevance of GNG4 as a therapeutic target for enhancing enzalutamide sensitivity in prostate cancer.

**Fig. 7:**
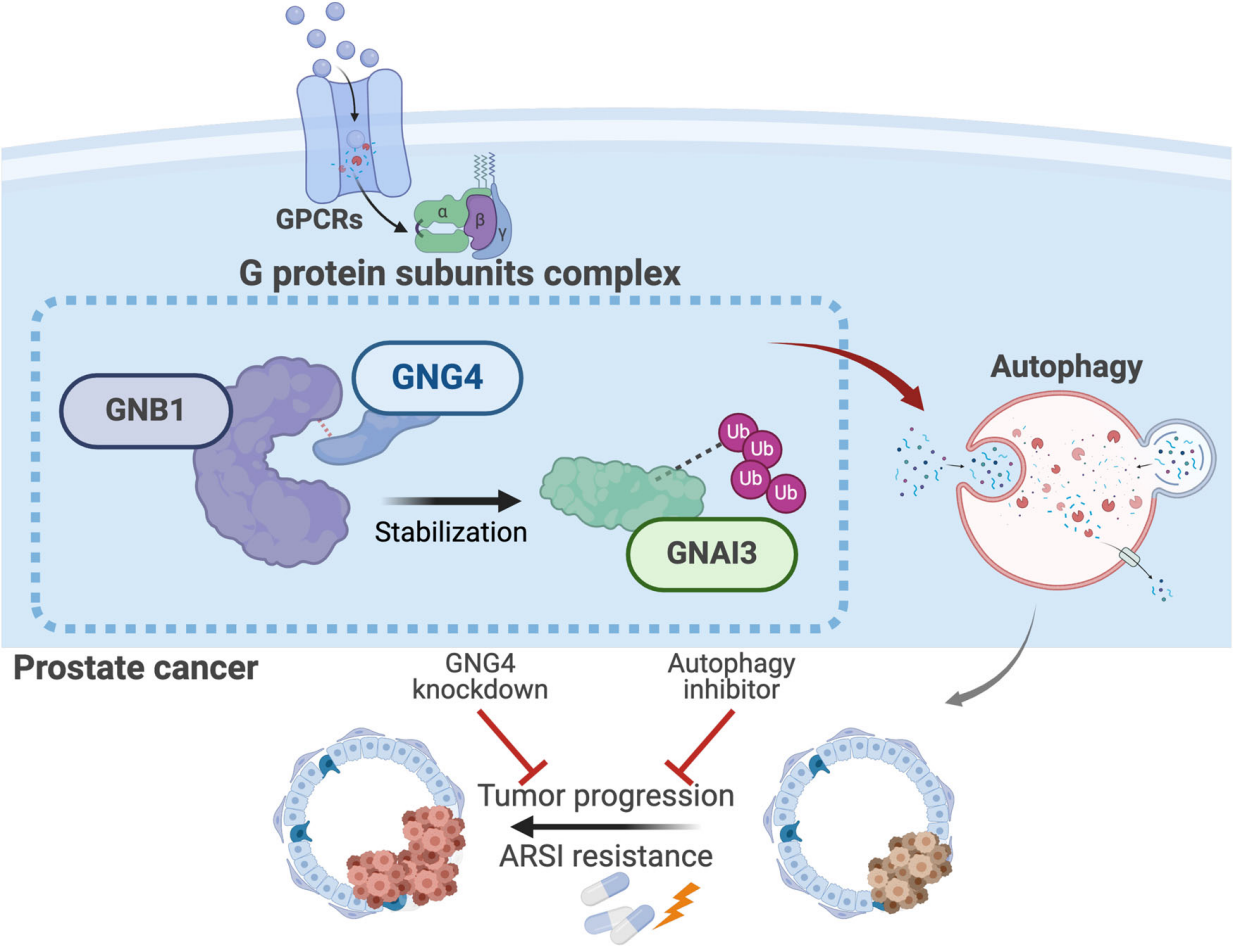
Schematic diagram delineating the mechanism by which GNG4 exerts its oncogenic function in prostate cancer. GNG4 interacts with GNB1 to stabilize GNAI3 by preventing its degradation through the ubiquitin–proteasome pathway. The stabilized GNAI3 promotes autophagy activation, leading to enhanced tumor cell proliferation and resistance to androgen receptor signaling inhibitors (ARSIs). GNG4 knockdown or autophagy inhibition suppresses these malignant effects and restores drug sensitivity.

In conclusion, our study identifies GNG4 as a regulator of PCa progression and treatment response. Autophagy, acting as a cytoprotective stress-response mechanism, underlies GNG4’s ability to promote cell survival and modulate drug sensitivity. We proposed a mechanistic model in which GNG4 forms a complex with other G protein subunits, such as GNB1 and GNAI3, stabilizing GNAI3 through the ubiquitin-proteasome pathway, thereby autophagy activation. Suppression of GNG4 disrupts this complex, leading to reduced autophagy and enhanced enzalutamide sensitivity. Collectively, these findings highlight GNG4 as a central mediator of enzalutamide resistance in PCa and indicate that underscore the translational relevance of GNG4 as a potential therapeutic target for improving the efficacy of androgen receptor–targeted therapies in PCa (Fig. 7).

## Discussion

Treating patients with advanced prostate cancer effectively continues to present a significant challenge, highlighting the urgent need to discover the possible oncogenic targets and develop novel therapeutic solutions. Researches focusing on G protein-coupled receptors (GPCRs), G proteins, and associated signaling pathways have emerged as one of the most promising strategies in cancer therapy. GPCRs, often referred to as 7TM receptors due to their structure of seven transmembrane helices, are the largest group of receptors located on cell surfaces and are a key target class of many drugs currently on the market [37, 38]. As integral membrane proteins, GPCRs respond to a broad range of activating agents, including photons, lipids, and proteins. In classical signaling pathways, ligand-activated GPCRs bind to G proteins, which transduce and amplify reactions via secondary messengers to produce downstream responses [39]. Signal transduction is primarily accomplished by activating the G protein heterotrimer, which consists of alpha, beta, and gamma subunits [40]. Upon stimulation by extracellular signals, the three subunits tightly assemble into trimeric structures. Due to their essential role in controlling various biological activities under both healthy and diseased conditions, an increasing number of studies have started to focus on the relationship between G proteins and cancer progression [41].

Although existing research have pointed to GNG4 as a potential oncogenic factor in several tumor types [16–19], the specific functions of GNG4 in PCa remain unknown. Our findings indicate that *GNG4* plays a crucial role as an oncogene in PCa. We observed GNG4 accumulation among higher-grade tumor types, which is not only related to numerous crucial signaling pathways involved in tumorigenesis but also significantly influences malignant progression, including cell cycle, proliferation, apoptosis, migration, and drug response. Our study highlights the critical involvement of GNG4 in PCa, proposing a novel treatment option aimed at inhibiting its activity. A previous study identified GNG4 as a key factor causing liver metastasis of gastric cancer [17]. It also functions in conjunction with type IV collagen via the PI3K/Akt and ERK signaling, helping tumor cells resist apoptosis and enhance their adhesion within the tumor microenvironment. Similarly, the risk of distant metastasis of PCa significantly increases with high-grade cancer, which may also be attributed to the accumulation of GNG4. We found that GNG4 is linked to multiple signaling pathways involved in tumorigenesis (Fig. 1E, F) and confirmed its effect of accelerating tumor migration (Fig. 2E, F). Additionally, a study conducted on bladder cancer has demonstrated that GNG4 is overexpressed in depleted CD4^+^ T cells, allowing tumor cells to evade immune recognition and further leading to the establishment of an immune suppressed microenvironment [20]. PCa is characterized as an immunologically “cold” tumor due to its low infiltration of immune cells and its limited response to immune checkpoint therapy [42]. One of the limitations of our study is that we did not conduct an in-depth investigation into the influence of GNG4 on the immune landscape of PCa. In summary, GNG4 represents a potential breakthrough for developing innovative therapeutic and diagnostic strategies.

Most patients with advanced prostate cancer still experience unfavorable therapeutic outcomes, including recurrence and therapy resistance, even after androgen deprivation therapy, posing a major challenge in clinical practice. Cancer cells evolve and progress through a variety of molecular mechanisms. Autophagy, a self-degradation process, facilitates the removal of damaged or aged cellular components to maintain homeostasis and regenerate energy [43]. Researchers have paid increasing attention to autophagy as a means to enhance the efficacy of tumor therapy [34]. Recent studies have demonstrated that PCa cells undergoing androgen deprivation, AR knockdown, or treatment with ARSIs exhibit an increased level of autophagy [27, 28]. The cancer cells modulate apoptosis via autophagy, promoting tumor progression. Similar to prior investigations, our data support the idea that GNG4 affects autophagy levels in PCa, thereby influencing tumor proliferation, survival, cell cycle, and metastatic potential. Additionally, we tried to explore whether GNG4 can influence the responsiveness to enzalutamide. The results indicate that PCa cells exploit autophagy, regulated by GNG4, as a survival mechanism to evade enzalutamide treatment. Moreover, inhibition of autophagy with 3-MA effectively restored therapeutic sensitivity. These findings demonstrate that autophagy functions not only as a sophisticated process for maintaining cell survival but also as a critical contributor to drug resistance.

However, autophagy gets involved in tumor progression in a complex manner, exhibiting both beneficial and detrimental effects depending on the context. Diosgenin, a natural steroidal compound, has been reported to decrease the expression of NEDD4, an oncogenic promoter in PCa, by inducing autophagy, thereby stimulating apoptosis and inhibiting cell proliferation [44–46]. In this context, autophagy exhibits a tumor-suppressive function. Conversely, previous studies by Mortezavi et al. and Feng et al. both demonstrated that basal autophagy are elevated in advanced and castration-resistant PCa cells [47, 48]. Moreover, irrespective of whether the ARSI abiraterone is administered alone or in combination with other agents, it consistently enhances intracellular autophagy activity. However, Ma et al. reported opposite findings, indicating that treatment with abiraterone decreases the levels of autophagy-associated proteins such as LC3, ATG5, and Beclin-1 [49]. Notably, these studies have demonstrated that inhibition of autophagic flux exerts significant tumor-suppressing effects. Furthermore, to enhance therapeutic efficacy, bioengineered nanoparticles encapsulating various antitumor agents have been developed to specifically target the autophagy pathway, thereby improving drug delivery and treatment outcomes [50–52]. Collectively, these findings suggest that autophagy plays a cytoprotective role in PCa, and elucidating the mechanisms underlying autophagy regulation is essential for improving the prognosis and survival of patients.

Previous studies have identified a diverse range of molecules and signaling networks, including non-coding RNAs, AMPK/PI3K, and mTOR pathways, as key players in the complex process of autophagy [34, 53]. However, the precise mechanism by which G proteins act as molecular “switches” to regulate autophagy in PCa remains unclear. In this study, we have elucidated how GNG4 enhances the sensitivity of PCa cells to enzalutamide and promotes tumor progression by suppressing autophagy. Through systematic screening and analyzing of autophagy-related proteins, we identified GNAI3 as a downstream effector regulated by GNG4. Our findings further revealed that GNG4 interacts with the mediator GNB1 to facilitate the degradation of GNAI3 through the ubiquitin-proteasome system, thereby modulating autophagy levels.

GNAI3, a key member of the GNAI protein family, exhibits diverse functional activities across multiple tissues [54], such as participating in vascular smooth muscle contraction, and orchestrating asymmetric cell division during embryonic development [55, 56]. In hepatocytes undergoing starvation-induced autophagy, the expression of GNAI3 is upregulated, accompanied by the accumulation of LC3 within both autophagosomes and lysosomes [35]. In addition, dysregulation of GNAI3 has been implicated in various types of tumors. GNAI3 is abundantly expressed in lung adenocarcinoma [57], accelerating the growth and metastatic spread of HeLa cells and glioblastoma multiforme [58, 59], contributing to poor clinical outcomes. In contrast to these findings, Zhang et al. reported that miR-222 negatively regulates GNAI3 expression in hepatocellular carcinoma, thereby facilitating tumor migration and metastasis [60]. While the involvement of the GNAI protein family in cancer progression has been gradually uncovered, detailed functional studies of GNAI3, particularly its involvement in PCa remain limited. Our findings demonstrated the function of GNAI3 as a promoter of PCa progression (Fig. 5) and plays an indispensable role in GNG4-mediated regulation of autophagy, highlighting GNAI3 as a novel contributor to PCa development.

Nevertheless, several limitations should be acknowledged in this study. First, although our results demonstrate that the protein GNG4/GNB1/GNAI3 constitute a functional trimer complex capable of regulating autophagy, further investigations are needed to determine whether this complex can be activated by upstream GPCRs and whether it possesses intrinsic downstream signaling capability. Second, while we provide preclinical evidence supporting autophagy modulation as a potential therapeutic strategy, it remains unsolved whether the dual roles of autophagy in facilitating both cell survival and death could serve as a possible breakthrough for improving treatment outcomes in patients with advanced PCa. Moreover, drugs that strongly suppress autophagy may not be well-tolerated by patients. Further research on treatment strategies of combining autophagy modulators that are both well-tolerated and safe with the ARSI therapy is necessary.

In conclusion, this study is the first to identify GNG4 as a key driver of PCa progression. We further uncovered a functional protein complex composed of GNG4, GNB1, and GNAI3 that regulates autophagy, thereby modulating influencing cellular behaviors including proliferation, migration, and drug sensitivity, particularly to enzalutamide. Overall, our findings highlight GNG4 as a potential research target for therapeutic intervention for patients with advanced PCa.

## Supplementary information

Supplemental Material

Original Data

## Data Availability

The dataset(s) supporting the findings of this study are included within the article. All other data supporting the findings of this study are available from the corresponding author upon reasonable request.
