# Supplementary material for: Targeting GNG4 inhibits tumor progression and restores enzalutamide sensitivity in prostate cancer by suppressing autophagy
Source: Cell Death Dis. 2026 Jan 28;17(1):160. doi: 10.1038/s41419-026-08421-w (PMC12877155; doi:10.1038/s41419-026-08421-w)
Supplement: Supplementary file 1 — Supplemental Material [file 41419_2026_8421_MOESM1_ESM.pdf]

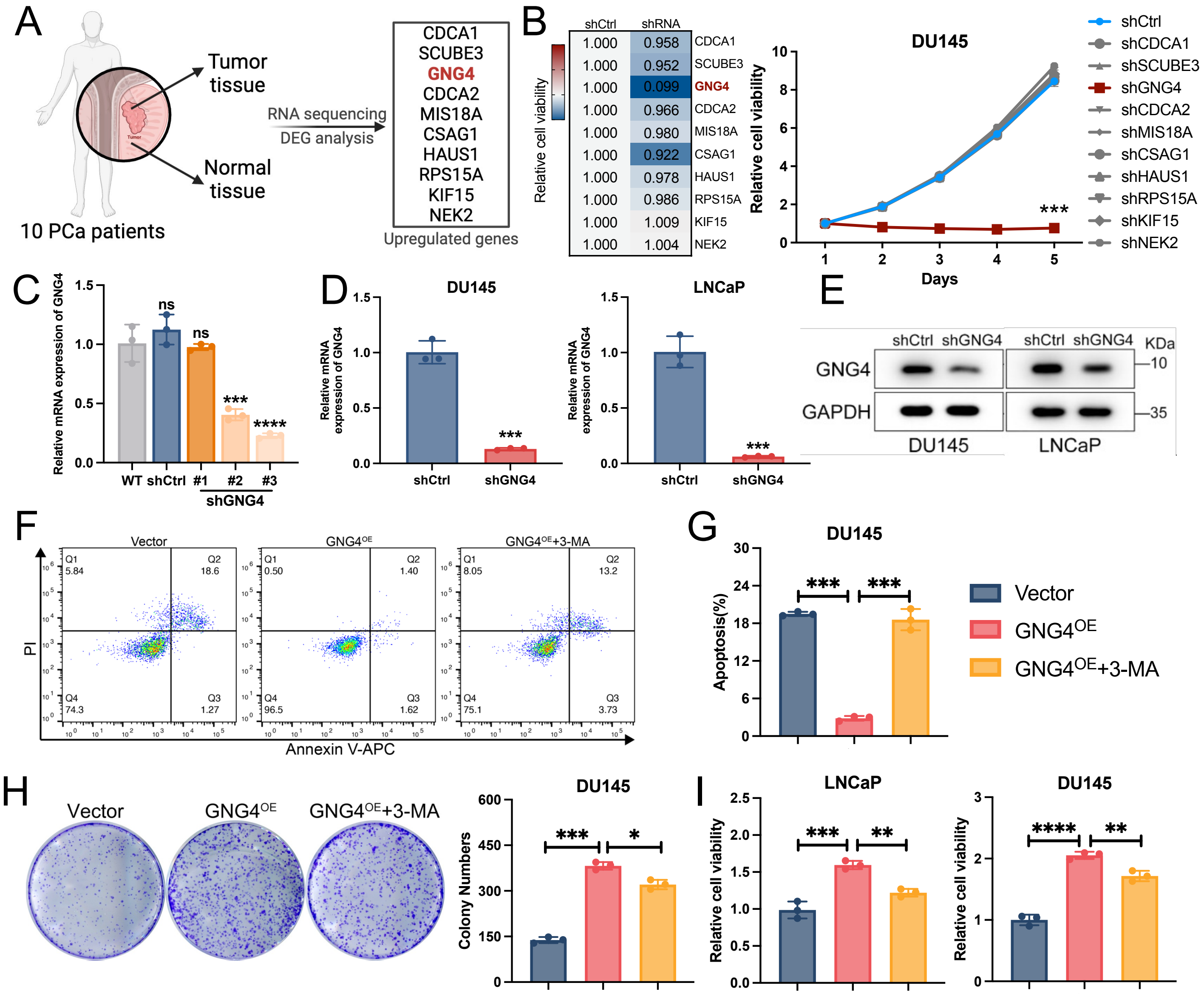

Supplementary Figure. 1

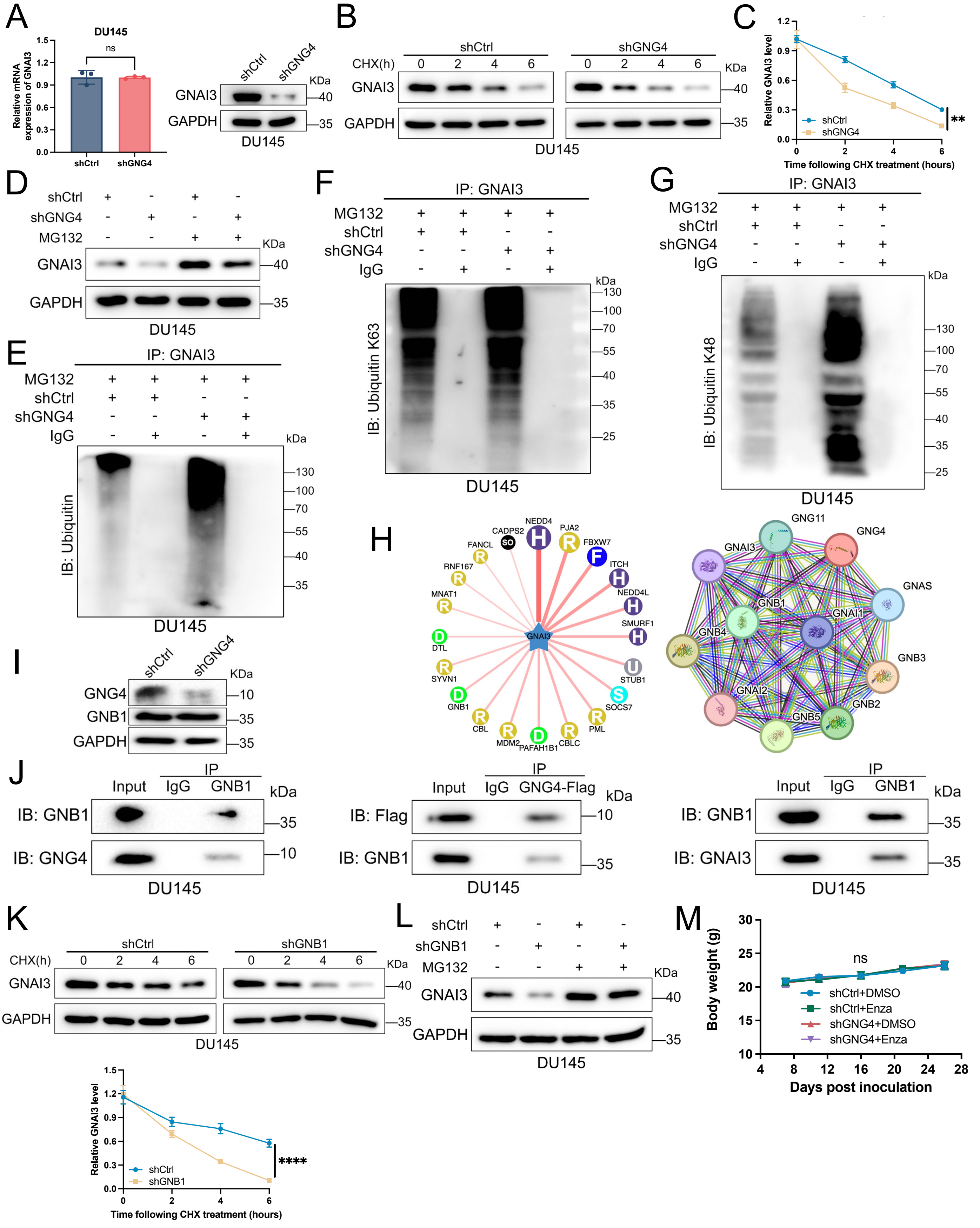

Supplementary Figure. 2

Supplementary Figure. 1

GNG4 deficiency inhibits autophagy level and cell proliferation in prostate cancer

(A) The flow chart illustrated the collection of tumor and adjacent normal tissues from 10 prostate cancer patients for transcriptome sequencing and further differential expression gene (DEG) analysis. The right panel highlighted several upregulated genes in tumor tissues. (B) Cell viability assays showed the impact of several genes knockdown in DU145 cells. The heatmap (left) demonstrated relative cell viability upon knockdown compared to the control. The CCK-8 assay depicted cell viability in 5 days (right). (C) DU145 cells were transfected with scramble negative control shRNA or three GNG4 shRNA and followed using qPCR analysis to examine the mRNA levels of GNG4. (D) mRNA level of GNG4 was measured by qPCR in DU145 and LNCaP cells treated with shCtrl or shGNG4. (E) Western blot assays were used to check the protein level of GNG4 in DU145 and LNCaP cells as indicated. Cell apoptosis level of DU145 cells transfected with shCtrl and shGNG4 was determined by flow cytometry. (F) Representative graph and (G) quantitative analysis. (H) Colony formation assays were performed to examine the cell proliferation of DU145 cells subjected to various treatments (Vector, $GNG4^{OE}$, $GNG4^{OE}$+3-MA). Representative photographs (left) and quantification bar chart (right) were shown. (I) Relative cell viability of LNCaP and DU145 with various treatment were determined by CCK-8 assay.

Supplementary Figure. 2

GNG4 and GNB1 stabilize GNAI3 protein by ubiquitination proteasome system

(A) mRNA level of GNAI3 between shCtrl and shGNG4 group in DU145 cells were examined by qPCR analysis. And western blot assay was used to determine the GNAI3 protein level after GNG4 knockdown. (B) DU145 cells stably knockdown GNG4 or negative control were treated with cycloheximide (CHX, 50μg/ml) at indicated time points by western blot analysis. (C) The GNAI3 protein expression level was quantified, and the plot was presented. (D) DU145 cells with shCtrl or shGNG4 were treated with 10μmol/L MG132 for 4h, the GNAI3 protein level was detected by western blot analysis. (E) Western blot analysis of ubiquitinated GNAI3 immunoprecipitated from DU145 cells with or without GNG4 knockdown. (F) Western blot analysis of K63-ubiquitinated GNAI3 immunoprecipitated from DU145 cells with or without GNG4 knockdown. (G) Western blot analysis of K48-ubiquitinated GNAI3 immunoprecipitated from LNCaP cells with or without GNG4 knockdown. (H) The predicted E3 ubiquitin ligase candidates of GNAI3 from Ubibrowser (left). Network diagram showing the potential proteins that interact with GNG4 from STRING database (right). (I) GNG4 and GNB1protein levels of shCtrl or shGNG4 in DU145 cells were measured with western blot analysis. (J) The interaction between GNG4 and GNB1 using anti-GNB1 antibody in DU145 cells was examined by immunoprecipitation (left). DU145 cells were transfected Flag-tagged GNG4 as indicated. Cell lysates were immunoprecipitated with Flag antibodies, and cell immunoprecipitates were immunoblotted with Flag or GNB1 antibodies (middle). The interaction between GNB1 and GNAI3 using anti-GNB1 antibody was examined by immunoprecipitation (right). (K) The GNAI3 protein expression level of DU145 cells stably knockdown GNB1 or negative control were treated with CHX at indicated time points by western blot analysis, and the plot of quantified protein level was presented. (L) DU145 cells with shCtrl or shGNB1 were treated with MG132, and the GNAI3 protein level was checked by western blot analysis. (M) The graph illustrated the average body weight of tumor-bearing mice.

Supplementary Table 1. Expression patterns in prostate cancer tumor tissues and paracancerous tissues revealed in immunohistochemistry analysis.

GNG4 expression	Tumor tissue		Paracancerous tissue		*p* value
	cases	percentage	Cases	percentage	
Low	44	48.9%	35	70.0%	0.007
High	46	51.1%	15	30.0%	

Supplementary Table 2. Relationship between GNG4 expression and tumor characteristics in patients with prostate cancer.

Features	No.	GNG4 expression		*p* value
		Low	High	
All patients	90	44	46	
Age (years)				0.823
≤ 71	48	24	24	
> 71	42	20	22	
Gleason Score				0.164
< 7	19	12	7	
≥ 7	71	32	39	
Grade				0.047
I	19	12	7	
II	40	20	20	
III	20	10	10	
IV	6	2	4	
V	5	0	5	

Supplementary Table 3. Spearman's rank correlation analysis of GNG4 expression and tumor grade in prostate cancer patients.

		GNG4
Grade	Spearman's	0.210
	p value (two-tailed test)	0.047*
	N	90

Supplementary File. List of antibodies used in this project

Name	Vendor	Catalog	Host	Dilution
GNG4	Invitrogen	PA5-68205	Rabbit	1:3000
GNAI3	abclonal	A13307	Rabbit	1:1000
GNB1	Proteintech	10247-2-AP	Rabbit	1:4000
LC3A/B	Proteintech	14600-1-AP	Rabbit	1:2000
P62	Proteintech	18420-1-AP	Rabbit	1:2000
GAPDH	Proteintech	60004-1-lg	Mouse	1:30000
HA tag	Proteintech	51064-2-AP	Rabbit	1:50/1:3000
DYKDDDDK tag	Proteintech	80010-1-RR	Rabbit	1:50/1:3000
Ubiquitin	Santa Cruz	sc-8017	Mouse	1:2000
Goat Anti-Rabbit	Beyotime	A0208		1:3000
Goat Anti-Mouse	Beyotime	A0216		1:3000
Ki67	abcam	ab16667	Rabbit	1:200

Corresponding Author Name: Chun-Wu Pan
Manuscript Number: CDDIS-25-3016R

SPRINGER NATURE

Reporting Summary

Springer Nature wishes to improve the reproducibility of the work that we publish. This checklist is used to ensure good reporting standards and to improve the reproducibility. Please respond completely to all questions relevant to your manuscript. For more information, please read the journal's Guide to Authors.

☑ **Check here to confirm that the following information is available in the Material & Methods section:**

- **The exact sample size (*n*)** for each experimental group/condition, given as a number, not a range
- **A description of the sample collection** allowing the reader to understand whether the samples represent technical or biological replicates (including how many animals, litters, culture, etc.)
- **A statement of how many times the experiment shown was replicated in the laboratory**
- **Definitions of statistical methods and measures**: For small sample sizes (n<5) descriptive statistics are not appropriate, instead plot individual data points
  - Very common tests, such as *t*-test, simple χ^2 tests, Wilcoxon and Mann-Whitney tests, can be unambiguously identified by name only, but more complex techniques should be described in the methods section
  - Are tests one-sided or two-sided?
  - Are there adjustments for multiple comparisons?
  - **Statistical test results**, e.g., ***P* values**
  - Definition of **'center values'** as **median or mean**;
  - Definition of **error bars as s.d. or s.e.m. or c.i.**

Please ensure that the answers to the following questions are reported in the manuscript itself. We encourage you to include a specific subsection in the methods section for statistics, reagents and animal models. Below, provide the page number or section and paragraph number.

Statistics and general methods

	**Reported in section/paragraph or page #**
1. How was the sample size chosen to ensure adequate power to detect a pre-specified effect size? (Give section/paragraph or page #)	Section Materials and methods: Xenograft Model in Nude Mice,Ethics statement
For animal studies, include a statement about sample size estimate even if no statistical methods were used.	Section Materials and methods: Xenograft Model in Nude Mice,Ethics statement
2. Describe inclusion/exclusion criteria if samples or animals were excluded from the analysis. Were the criteria pre-established? (Give section/paragraph or page #)	Section Materials and methods: Xenograft Model in Nude Mice,Ethics statement
3. If a method of randomization was used to determine how samples/animals were allocated to experimental groups and processed, describe it. (Give section/paragraph or page #)	Section Materials and methods: Xenograft Model in Nude Mice,Ethics statement
For animal studies, include a statement about randomization even if no randomization was used.	Section Materials and methods: Xenograft Model in Nude Mice,Ethics statement

4. If the investigator was blinded to the group allocation during the experiment and/or when assessing the outcome, state the extent of blinding. (Give section/paragraph or page #)	Section Materials and methods: Xenograft Model in Nude Mice,Ethics statement
For animal studies, include a statement about blinding even if no blinding was done.	Section Materials and methods: Xenograft Model in Nude Mice,Ethics statement
5. For every figure, are statistical tests justified as appropriate?	Yes.<br>Section Materials and methods: Statistical analysis
Do the data meet the assumptions of the tests (e.g., normal distribution)?	Yes.<br>Section Materials and methods: Statistical analysis
Is there an estimate of variation within each group of data?	Yes.<br>Section Materials and methods: Statistical analysis
Is the variance similar between the groups that are being statistically compared? (Give section/paragraph or page #)	Yes.<br>Section Materials and methods: Statistical analysis

Reagents	**Reported in section/paragraph or page #**
6. Report the source of antibodies (vendor and catalog number)	See in the File: Supplementary table_antibodies.
7. Identify the source of cell lines and report if they were recently authenticated (e.g., by STR profiling) and tested for mycoplasma contamination	Section Materials and methods: Cell culture, plasmids, and transfection

Animal Models	**Reported in section/paragraph or page #**
8. Report species, strain, sex and age of animals	Section Materials and methods: Xenograft Model in Nude Mice.
9. For experiments involving live vertebrates, include a statement of compliance with ethical regulations and identify the committee(s) approving the experiments.	Section Materials and methods: Xenograft Model in Nude Mice,Ethics statement

10. We recommend consulting the ARRIVE guidelines (*PLoS Biol.* **8**(6), e1000412,2010) to ensure that other relevant aspects of animal studies are adequately reported.

Human subjects

	Reported in section/paragraph or page #
11. Identify the committee(s) approving the study protocol.	NA
12. Include a statement confirming that informed consent was obtained from all subjects.	NA
13. For publication of patient photos, include a statement confirming that consent to publish was obtained.	NA
14. Report the clinical trial registration number (at ClinicalTrials.gov or equivalent).	NA

15. For phase II and III randomized controlled trials, please refer to the CONSORT statement and submit the CONSORT checklist with your submission.

16. For tumor marker prognostic studies, we recommend that you follow the REMARK reporting guidelines.

Data deposition

	Reported in section/paragraph or page #
17. Provide accession codes for deposited data. Data deposition in a public repository is mandatory for:<br>a. Protein, DNA and RNA sequences<br>b. Macromolecular structures<br>c. Crystallographic data for small molecules<br>d. Microarray data	NA

Deposition is strongly recommended for many other datasets for which structured public repositories exist; more details on our data policy are available in the Guide to Authors. We encourage the provision of other source data in supplementary information or in unstructured repositories such as Figshare and Dryad. We encourage publication of Data Descriptors (see Scientific Data) to maximize data reuse.

18. If computer code was used to generate results that are central to the paper's conclusions, include a statement in the Methods section under "**Code availability**" to indicate whether and how the code can be accessed. Include version information as necessary and any restrictions on availability.	NA
