# Supplementary material for: Targeting GNG4 inhibits tumor progression and restores enzalutamide sensitivity in prostate cancer by suppressing autophagy
Source: Cell Death Dis. 2026 Jan 28;17(1):160. doi: 10.1038/s41419-026-08421-w (PMC12877155; doi:10.1038/s41419-026-08421-w)

Fig. 1E

WPMY1	DU145	LNCaP
0.998294659	3.102639157	13.89037682
1.005096979	3.149651866	14.30677765
0.99662846	3.343583153	14.89116987

Fig. 4C

shCtrl

0.992	1.047	0.963

shGNG4

0.991	1.02	0.987

Fig. S2A

shCtrl

1.049	1.062	0.898

shGNG4

0.983	1.003	1.015

Fig. S1C

		shGNG4		
WT	shCtrl	#1	#2	#3
1.150866367	1.126102198	0.981897879	0.360429449	0.205760326
0.838788264	0.997162018	1.000634576	0.455667162	0.246535553
1.035911744	1.250982515	0.948463874	0.397396027	0.230776905

Fig. S1D

DU145

shCtrl	shGNG4
1.1223792	0.13719168
0.945037	0.13674875
0.94278264	0.11993002

LNCaP

shCtrl	shGNG4
1.161610084	0.066851401
0.883095413	0.054077613
0.974836985	0.064892854

Fig.3 C

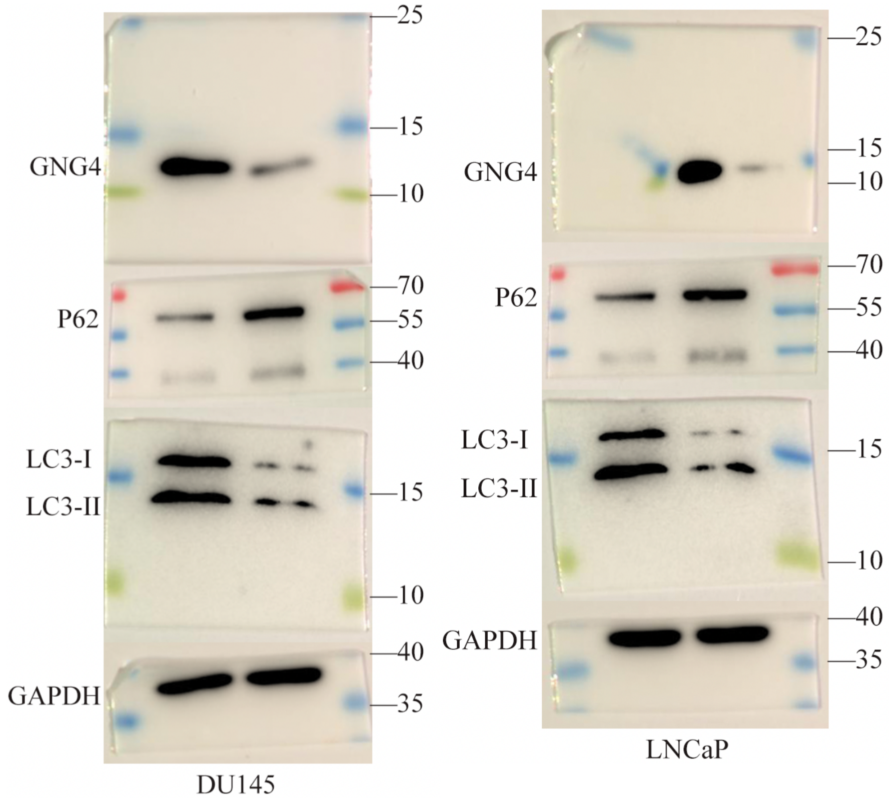

GNG4
P62
LC3-I
LC3-II
GAPDH
25
15
10
70
55
40
15
10
40
35
DU145
GNG4
P62
LC3-I
LC3-II
GAPDH
25
15
10
70
55
40
15
10
40
35
LNCaP

Fig.4 B

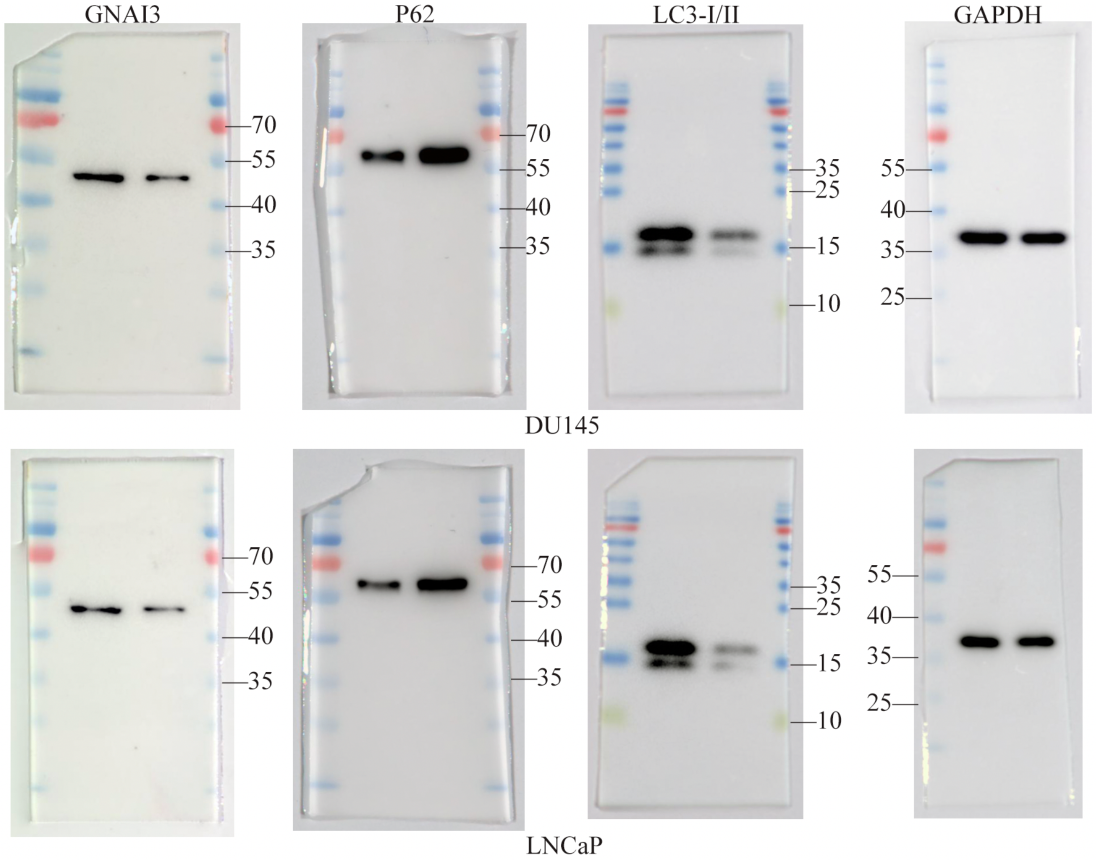

GNAI3
P62
LC3-I/II
GAPDH
70
55
40
35
70
55
40
35
35
25
15
10
55
40
35
25
DU145
70
55
40
35
70
55
40
35
35
25
15
10
55
40
35
25
LNCaP

Fig.4 C

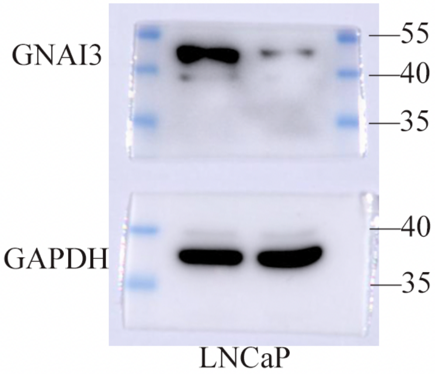

Fig.4 D

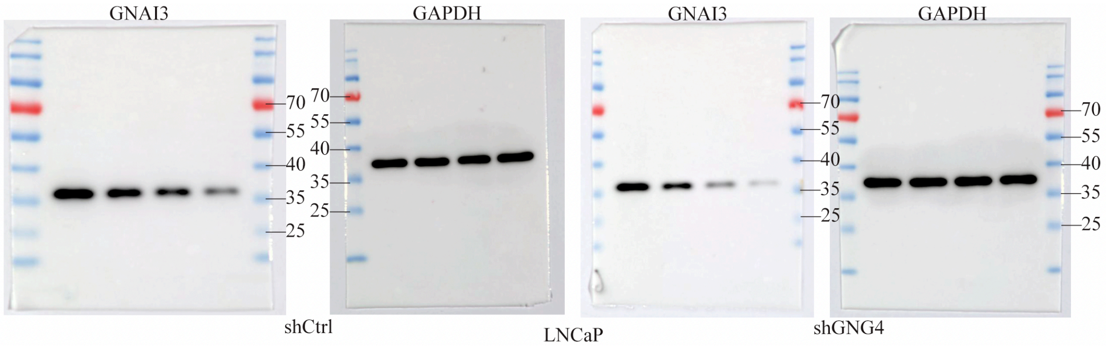

Fig.4 E

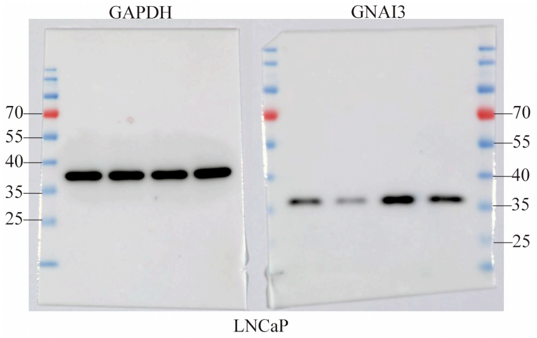

Fig.4 F

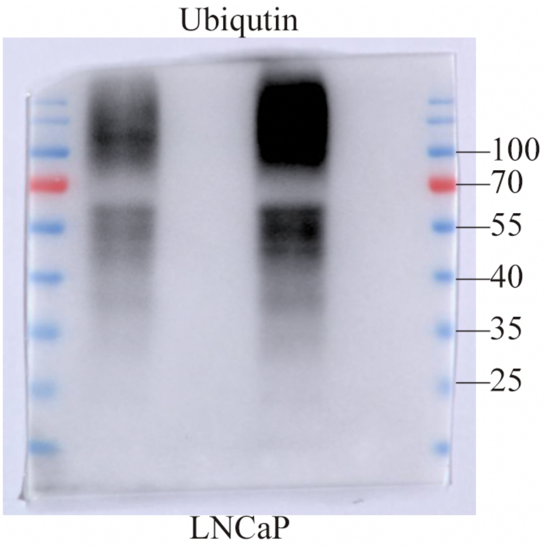

Fig.4 G

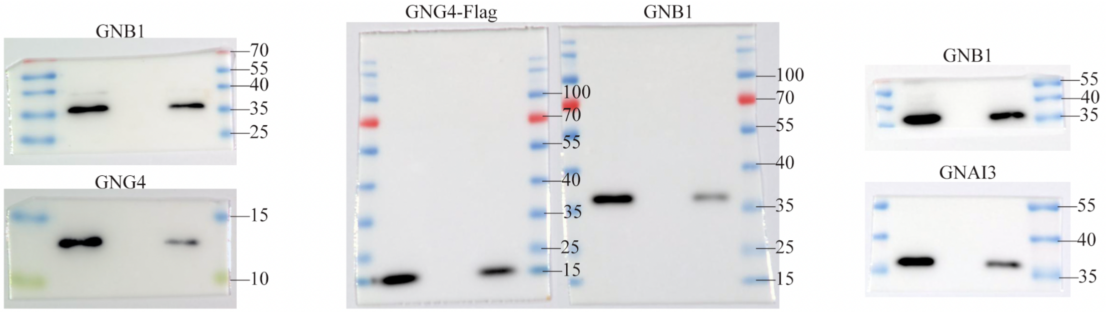

Fig.4 J

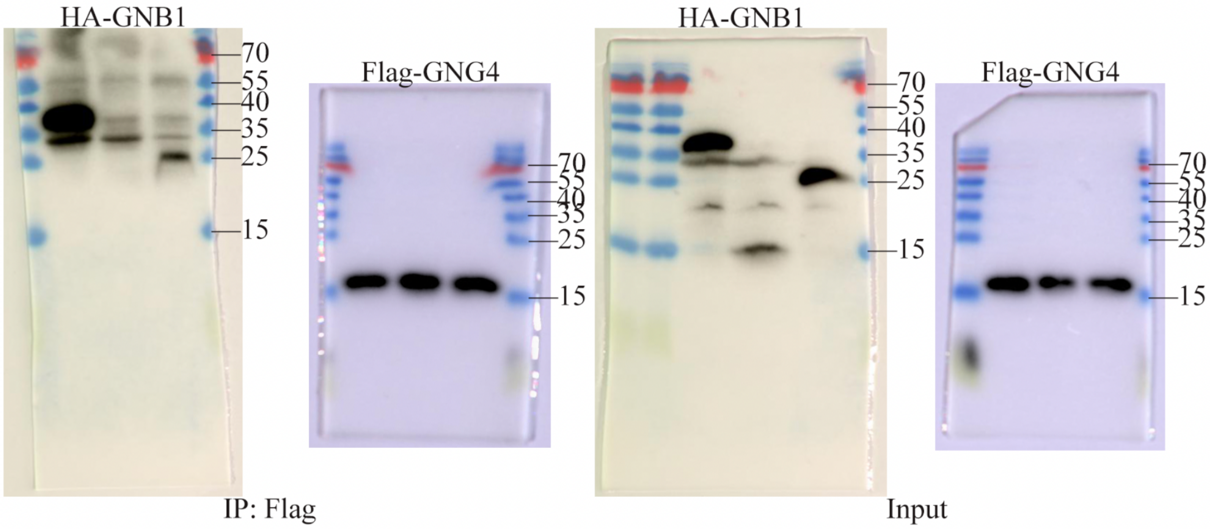

Fig.4 K

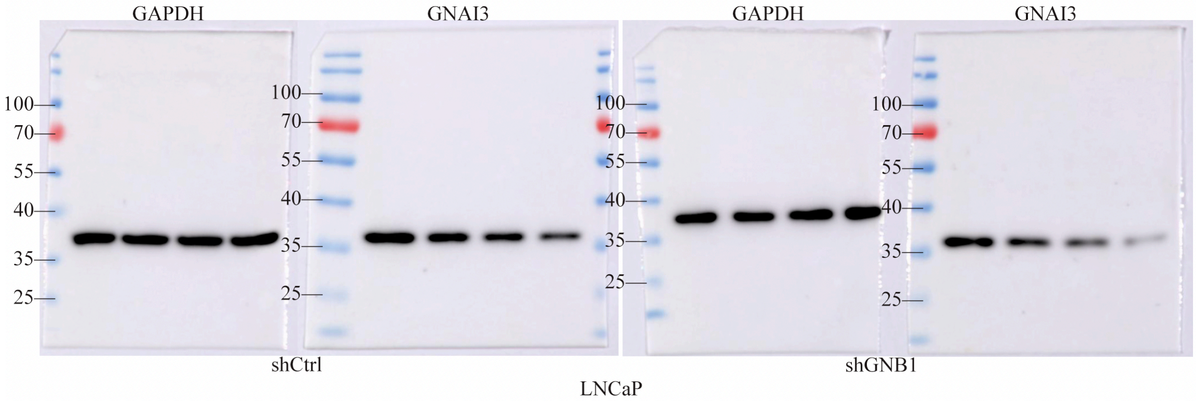

Fig.4 M

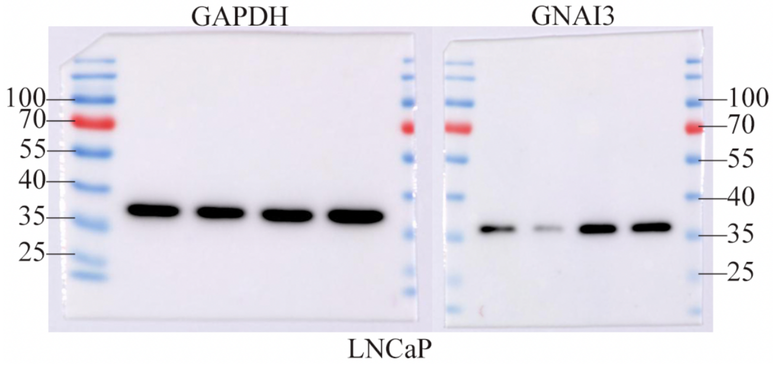

Fig.5 A

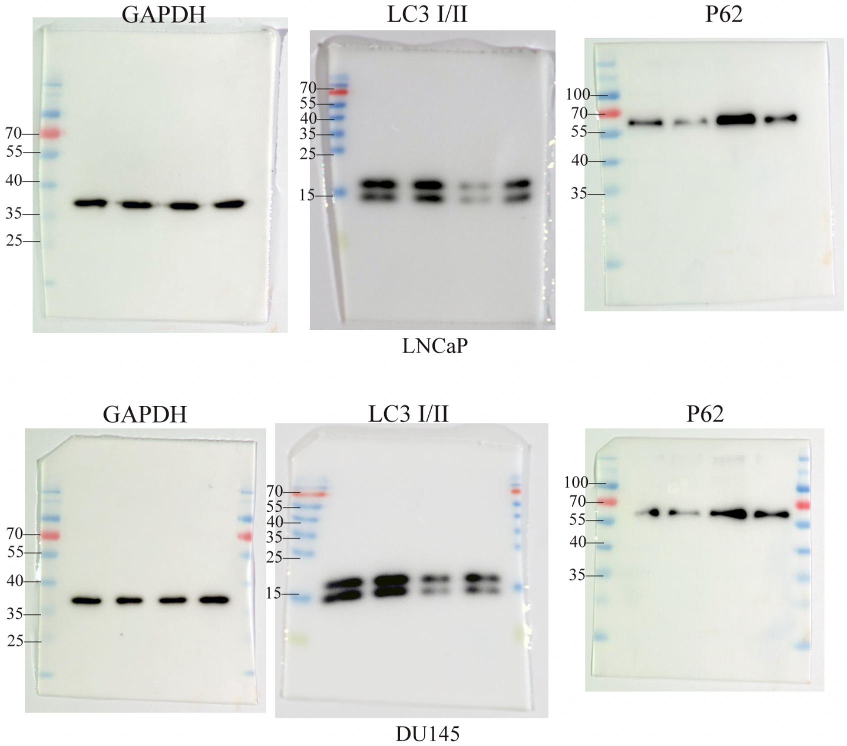
GAPDH
LC3 I/II
P62
LNCaP
GAPDH
LC3 I/II
P62
DU145

Fig.5 I

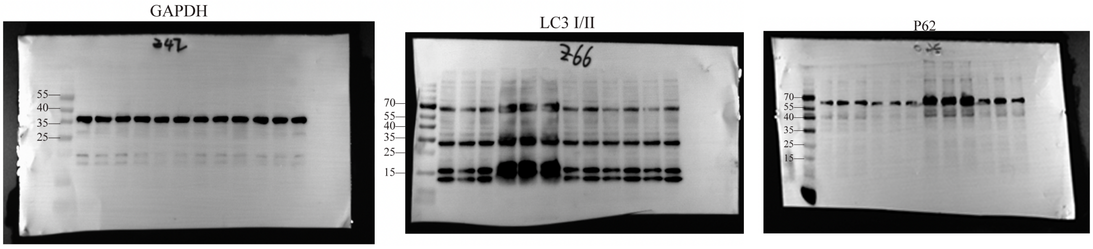
GAPDH
LC3 I/II
P62

Fig.6 A

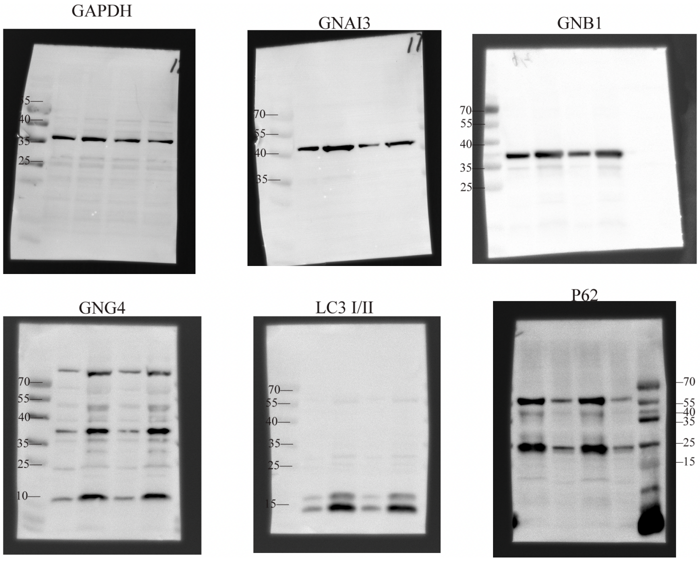
GAPDH
GNAI3
GNB1
GNG4
LC3 I/II
P62

Fig. 6L

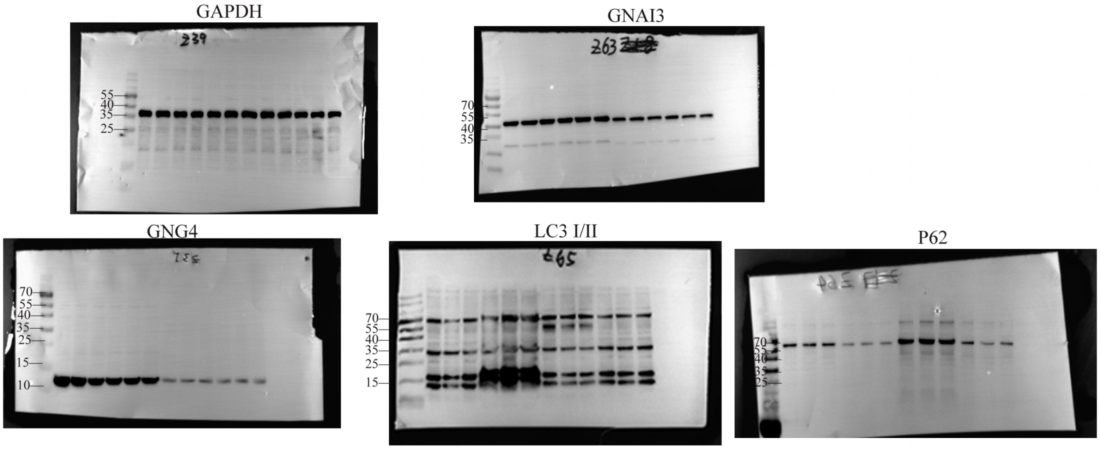
GAPDH
GNAI3
GNG4
LC3 I/II
P62

Supplementary Fig. 1 E

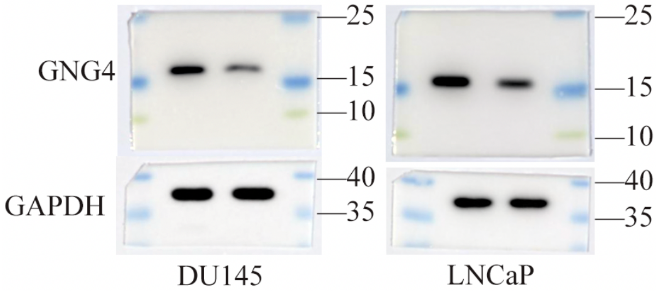

Supplementary Fig. 2 A

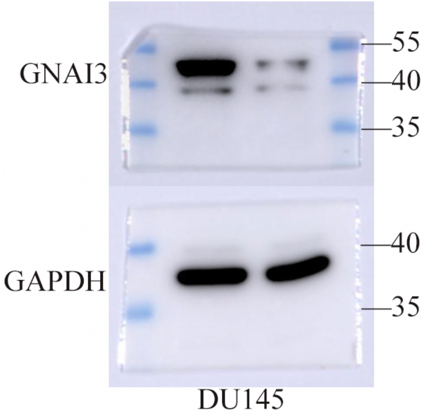

Supplementary Fig. 2 B

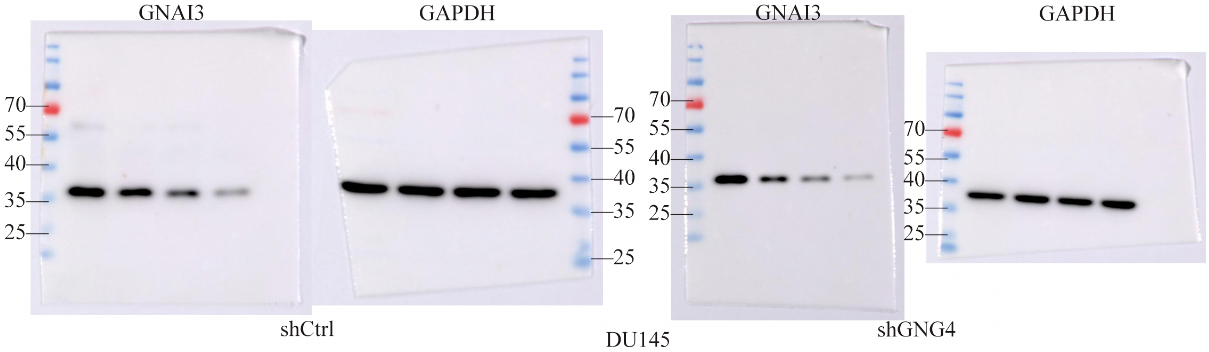

Supplementary Fig. 2 D

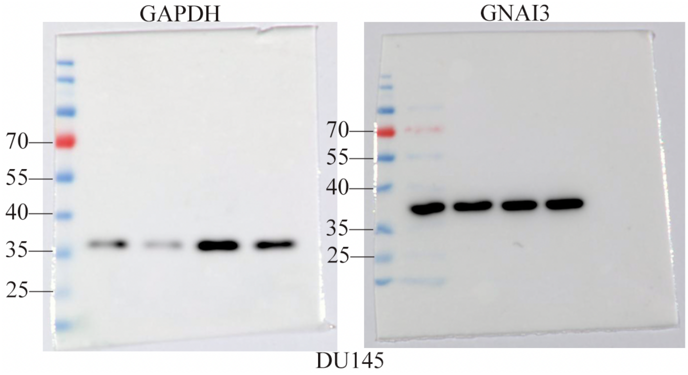

Supplementary Fig. 2 E

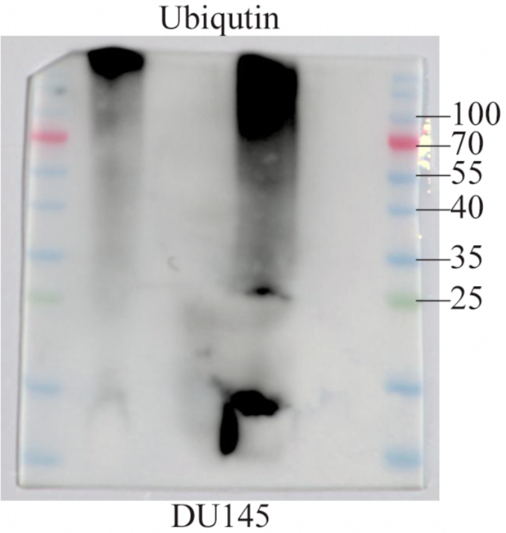

Supplementary Fig. 2 F & G

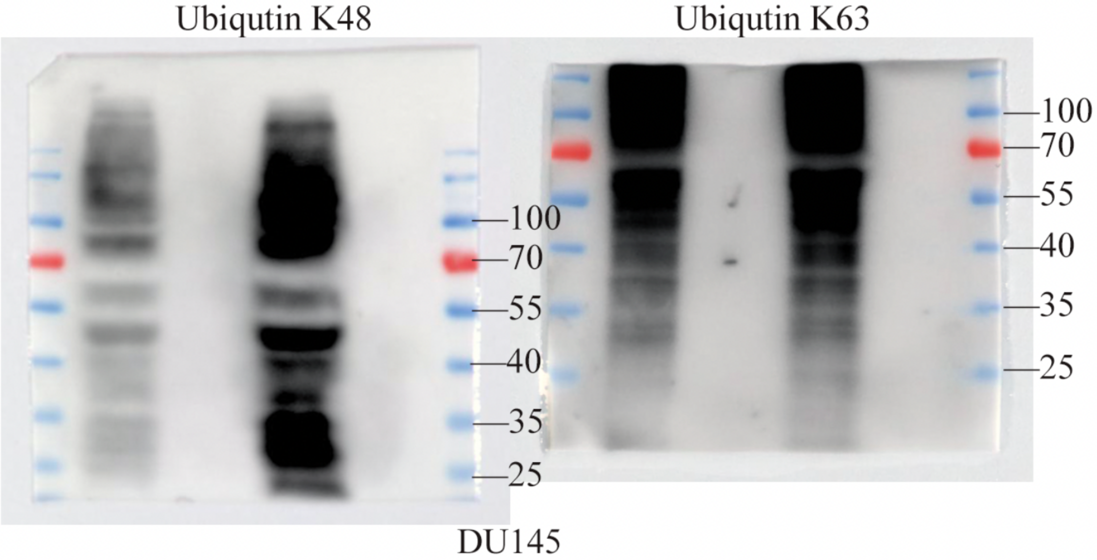

Supplementary Fig. 2 J

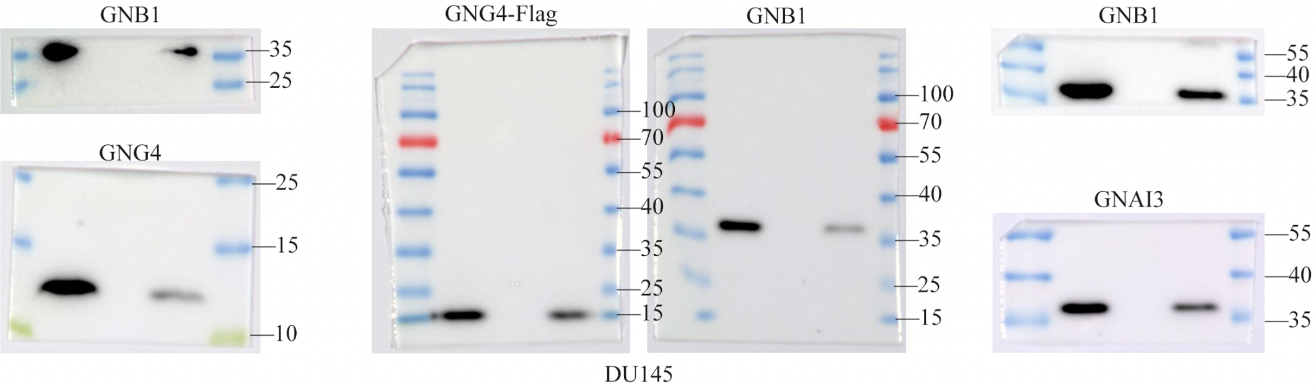

Supplementary Fig. 2 K

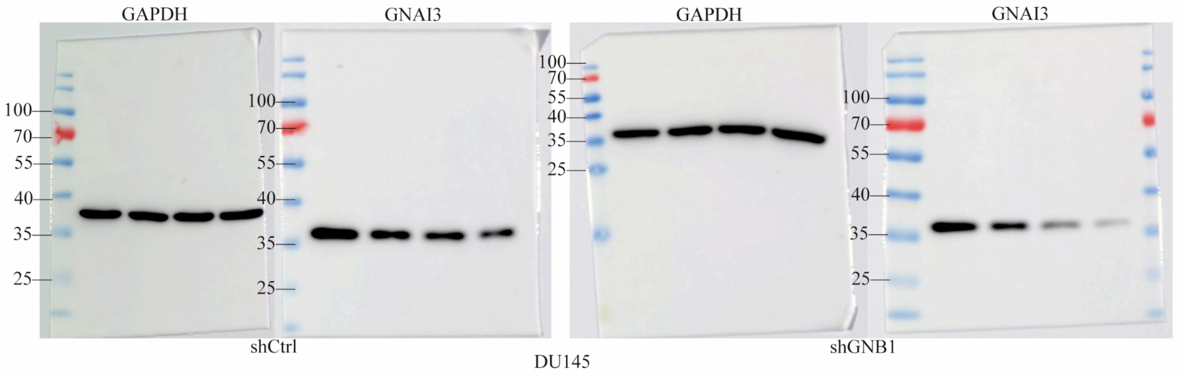

Supplementary Fig. 2 L

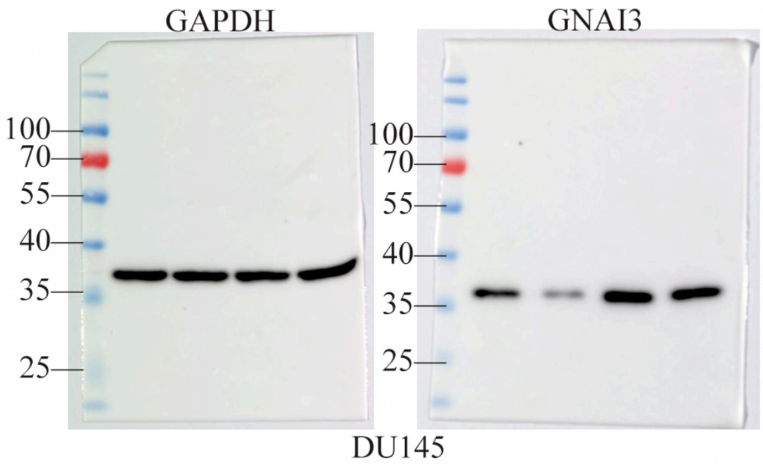

Supplement: Supplementary file 2 — Original Data [file 41419_2026_8421_MOESM2_ESM.pdf]
